# Comprehensive understanding of multiple resonance thermally activated delayed fluorescence through quantum chemistry calculations

**DOI:** 10.1038/s42004-022-00668-6

**Published:** 2022-04-14

**Authors:** Katsuyuki Shizu, Hironori Kaji

**Affiliations:** Institute for Chemical Research, Kyoto University, Uji, Kyoto 611-0011 Japan

**Keywords:** Electronic devices, Electronic materials, Excited states, Quantum chemistry

## Abstract

Molecules that exhibit multiple resonance (MR) type thermally activated delayed fluorescence (TADF) are highly efficient electroluminescent materials with narrow emission spectra. Despite their importance in various applications, the emission mechanism is still controversial. Here, a comprehensive understanding of the mechanism for a representative MR-TADF molecule (5,9-diphenyl-5,9-diaza-13b-boranaphtho[3,2,1-*de*]anthracene, DABNA-1) is presented. Using the equation-of-motion coupled-cluster singles and doubles method and Fermi’s golden rule, we quantitatively reproduced all rate constants relevant to the emission mechanism; prompt and delayed fluorescence, internal conversion (IC), intersystem crossing, and reverse intersystem crossing (RISC). In addition, the photoluminescence quantum yield and its prompt and delayed contributions were quantified by calculating the population kinetics of excited states and the transient photoluminescence decay curve. The calculations also revealed that TADF occurred via a stepwise process of 1) thermally activated IC from the electronically excited lowest triplet state T_1_ to the second-lowest triplet state T_2_, 2) RISC from T_2_ to the lowest excited singlet state S_1_, and 3) fluorescence from S_1_.

## Abstract (web summary)

Molecules that exhibit multiple resonance thermally activated delayed fluorescence (MR-TADF) are highly efficient electroluminescent materials, but their emission mechanism is still not fully understood. Here, the emission mechanism of a representative MR-TADF molecule is studied through quantum chemistry calculations, with two consecutive decay processes unveiled.

## Introduction

Since highly efficient thermally activated delayed fluorescence (TADF) was used in organic light-emitting diodes (OLEDs)^1^, many TADF molecules containing various electron donor and acceptor groups have been developed^2–9^. An important factor for TADF efficiency is the energy difference between the lowest triplet state (T_1_) and the lowest excited singlet state (S_1_), ∆*E*(T_1_ → S_1_); a small ∆*E*(T_1_ → S_1_) (<200 meV) is required to induce T_1_ → S_1_ reverse intersystem crossing (RISC). Another important factor is a large transition-dipole moment between S_1_ and ground-state S_0_ to accelerate the rate of S_1_ → S_0_ fluorescence. Experimentally, efficient RISC and high photoluminescence quantum yields (PLQYs) have been simultaneously realized by combining suitable donor and acceptor units that control the spatial overlap between the highest-occupied molecular orbitals (HOMOs) and the lowest-unoccupied molecular orbitals (LUMOs)^1–9^. It is important to note that the fluorescence spectra were broadened because of the charge-transfer character of S_1_, which was a significant drawback for OLED applications in displays.

**Fig. 1:**
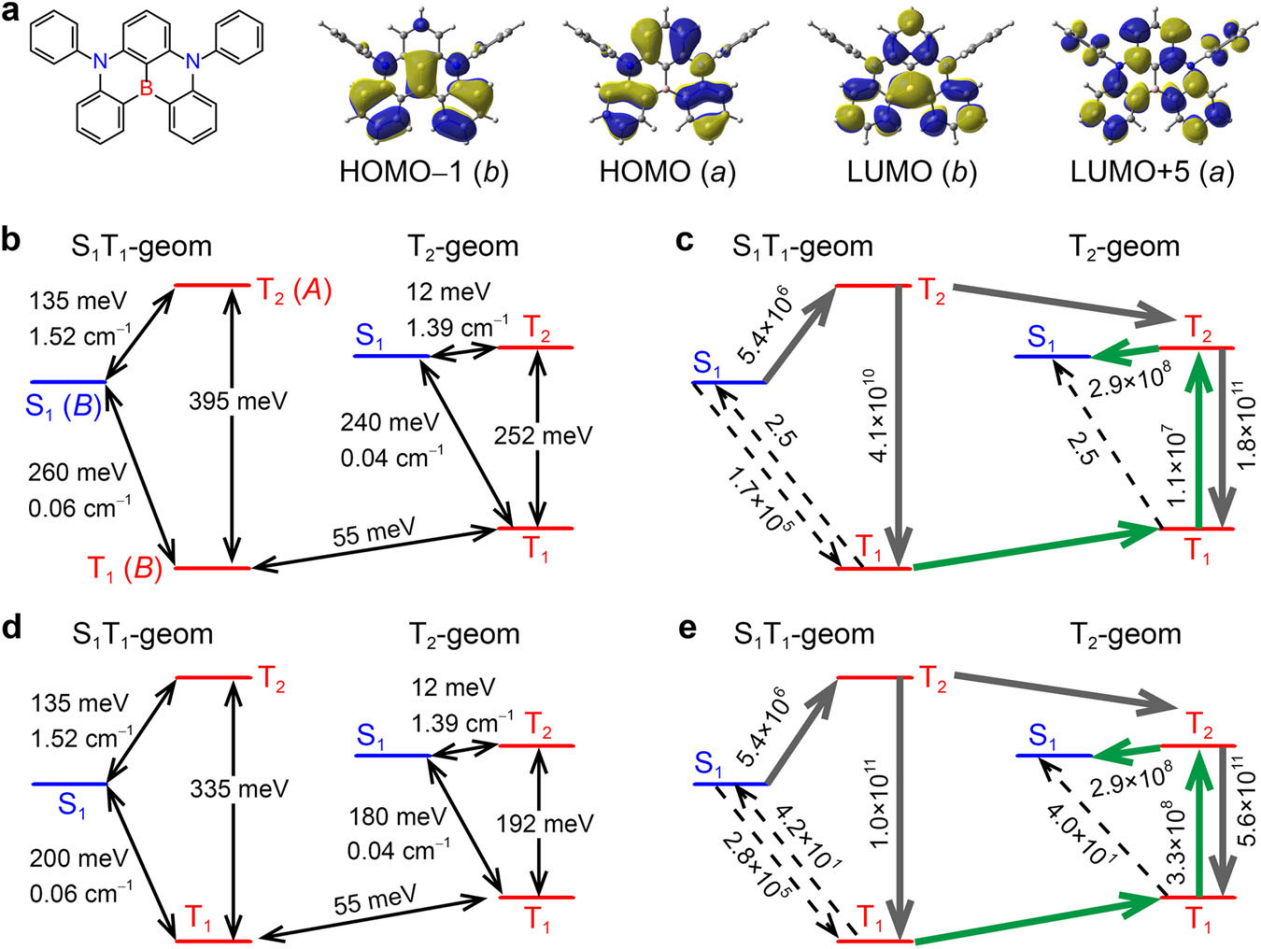
Molecular orbitals and electronic transitions of DABNA-1. **a** Structure of DABNA-1 and HOMO-1, HOMO, LUMO, and LUMO+5 distributions calculated at the RHF/6-31 G level (HOMO = highest-occupied molecular orbital, LUMO = lowest-unoccupied molecular orbital). The orbital symmetry is shown in parentheses. **b** Energy differences (meV) between S_1_, T_1_, and T_2_. S_1_–T_1_ and S_1_–T_2_ spin–orbit couplings (cm^−1^). The state symmetry is shown in parentheses. **c** The stepwise S_1_ → T_2_ → T_1_ and T_1_ → T_2_ → S_1_ processes of DABNA-1. The gray solid arrow depicts stepwise S_1_ → T_2_ → T_1_; the green solid arrow depicts stepwise T_1_ → T_2_ → S_1_; the dotted arrows show minor decay and upconversion processes. The values are calculated rate constants for one-step transitions. **d** The energy differences calculated using the corrected T_1_ energy. **e** Calculated rate constants for one-step transitions using the corrected T_1_ energy.

In 2016, Hatakeyama et al. developed a new class of TADF molecules^10^. Using a triphenylboron core possessing two nitrogen atoms (5,9-diphenyl-5,9-diaza-13b-boranaphtho[3,2,1-*de*]anthracene, DABNA-1, Fig. 1a), they observed a HOMO–LUMO separation without a conventional donor–acceptor structure, providing efficient TADF with a narrow emission spectrum. The HOMO–LUMO separation resulted from a multiple resonance (MR) effect, that is an opposite resonance effect induced by the boron and nitrogen atoms. MR–TADF molecules emitting blue-to-red fluorescence have been reported^11–39^.

Several theoretical studies have attempted to reveal the TADF mechanism in MR molecules. Northey et al. investigated an intersystem-crossing (ISC) mechanism in DABNA-1 using quantum dynamics and time-dependent density-functional theory (TD-DFT) at the PBE0/6-31 G(d) level^40^. There was only a 0.02-eV energy difference between T_2_ and S_1_, ∆*E*(T_2_ → S_1_). However, ∆*E*(T_1_ → S_1_) and ∆*E*(T_1_ → T_2_) were large, 0.59 eV and 0.61 eV, respectively, which could not explain efficient RISC from T_1_ to S_1_. Gao et al. examined the density-functional dependence of ∆*E*(T_1_ → S_1_) within the framework of TD-DFT^41^. The MPWK1CIS functional reproduced the experimental ∆*E*(T_1_ → S_1_)^41^, as reviewed by Suresh et al.^21^. However, the RISC rate constant (*k*_RISC_) was not calculated and the TADF mechanism was unclear. Also, they considered TADF in terms of direct (one-step) T_1_ → S_1_ RISC, which differed from the work of Northey et al^40^. Pershin et al. reported that TD-DFT methods overestimated ∆*E*(T_1_ → S_1_) for MR molecules, and that the spin-component-scaling second-order approximate coupled-cluster (SCS-CC2) method outperformed TD-DFT for predicting ∆*E*(T_1_ → S_1_)^42,43^. The partial inclusion of double excitations within the SCS-CC2 method was responsible for the improved accuracy in predicting ∆*E*(T_1_ → S_1_). The SCS-CC2 calculation of DABNA-1-based molecules revealed the relationship between its molecular structure and electronic properties, ∆*E*(T_1_ → S_1_), and the fluorescence rate constant. However, the TADF mechanism was still unclear because the *k*_RISC_ was not calculated. Lin et al. calculated rate constants for fluorescence (*k*_F_), ISC (*k*_ISC_), *k*_RISC_, and internal conversion (IC) (*k*_IC_) for DABNA-1^44^. However, the calculated *k*_RISC_ was 6.7 × 10^2 ^s^−1^, which was much less than the experimental value of 1.0 × 10^4^ s^−1^^10^. The calculated *k*_ISC_ of 1.4 × 10^4 ^s^−1^ was two orders of magnitude less than the experimental value of 4.5 × 10^6^ s^−1^^10^. In addition, the calculated nonradiative decay, *k*_IC_(S_1_ → S_0_), was greater than the *k*_F_(S_1_ → S_0_), suggesting that the PLQY of DABNA-1 was less than 50%, in contrast with the 88% experimental value^10^. All these previous studies^40–44^ only partially explained the photophysical properties of MR-TADF mainly because crucial RISC was not elucidated.

Here, we report a comprehensive understanding of the TADF mechanism using the representative MR molecule, DABNA-1. So far, ∆*E*(T_1_ → S_1_) (and spin–orbit coupling (SOC) in some cases) and oscillator strength have been considered to understand the TADF mechanism and to design TADF molecules. However, these are not sufficient for the above aim; quantifications of all types of rate constants and those of energy levels, including higher-lying states relevant to the emission process, are required for the complete understanding of the emission mechanism. Recently, we reported rate-constant predictions enabled by a proposed cost-effective calculation method based on Fermi’s golden rule^45^. Using the method, we calculated *k*_IC_, *k*_ISC_, *k*_RISC_, *k*_F_, and the rate constant for phosphorescence (*k*_Phos_), of benzophenone as an example. The calculated rate constants agreed well with experimental values. Here, we applied the same method to DABNA-1 and determined all relevant rate constants. We also determined the lifetime of the delayed component in transient PL (trPL), the rate constant of TADF (*k*_TADF_), and the PLQY, by calculating the population kinetics of the excited states and the trPL decay curve. The calculations also indicated that, after photoexcitation, T_1_ was first generated via S_1_ → T_2_ ISC and T_2_ → T_1_ IC. Then, triplet-to-singlet conversion occurred via thermally activated T_1_ → T_2_ IC and T_2_ → S_1_ RISC.

## Results and discussion

### Excited states of DABNA-1

S_0_, S_1_, T_1_, and T_2_ geometries of DABNA-1 were obtained at the TPSSh/6-31+G(d) level. Then, excited states were computed using the equation-of-motion coupled-cluster singles and doubles (EOM-CCSD) method (see “Methods” section). At the TD-TPSSh level, S_1_ and T_1_ local-energy minima were located at the S_1_- and T_1_-optimized geometries, respectively. However, as shown in Supplementary Table 10–12 and Supplementary Fig. 3, the lowest S_1_ and T_1_ energy levels calculated at the EOM-CCSD level were both located at the T_1_ geometry (3.37 and 3.12 eV, respectively). In addition, the S_1_ and T_1_ structures were nearly the same, including the dihedral angles (Supplementary Table 5), hence, the structure was denoted the S_1_T_1_ geometry. Because the Stokes shift was experimentally observed for DABNA-1 in solution and in solid films^10^, it was reasonably assumed that fluorescence occurs in  the S_1_ structure at the adiabatic energy minimum. The S_0_, S_1_, T_1_, and T_2_ electronic states and the S_1_T_1_ and T_2_ equilibrium geometries were used to model TADF for DABNA-1 (eight electronic states in all). Because S_2_ states were 0.89 eV higher in energy than the S_1_ states, their contributions were neglected (Supplementary Fig. 3). The T_3_ states were only 0.2 eV higher than S_1_, but were neglected because the electronic-transition rate constants from S_1_ to T_3_ were far smaller than those of the competing processes (S_1_–T_1_ and S_1_–T_2_) and that from T_2_ to T_3_ was smaller than that from T_2_ to T_1_ (Supplementary Fig. 3). In the EOM-CCSD calculations, S_1_ and T_1_ involved predominantly HOMO → LUMO transitions, whereas T_2_ involved predominantly a linear combination of HOMO → LUMO + 5 and HOMO − 1 → LUMO transitions (Fig. 1a, Supplementary Fig. 2, and Supplementary Tables 6–9). Figure 1b shows a calculated energy-level diagram of DABNA-1. The adiabatic T_1_ → S_1_ energy difference calculated with the EOM-CCSD/6-31 G method (S_1_T_1_ geometry) was only 0.06 eV larger than the experimentally obtained 0.2 eV. Thus, EOM-CCSD significantly improved the overestimation of ∆*E*(T_1_ → S_1_), relative to those by previously reported TD-DFT methods^40,42–44^.

### Calculation of rate constants and luminescence quantum efficiencies

We examined triplet formation from S_1_. Figure 1b shows calculated energy differences and SOCs, and Table 1 lists calculated and experimental values of *k*_IC_, *k*_ISC_, *k*_RISC_, *k*_F_, and *k*_Phos_. The raw rate constants in Table 1 were calculated using excitation energies calculated via EOM-CCSD. Corrected values are discussed below. Equations for *k*_IC_, *k*_ISC_, *k*_RISC_, *k*_F_, and *k*_Phos_ are given in Supplementary Equations S1–S9 and Supplementary Fig. 4.

**Table 1:** Calculated and experimental photophysical properties of DABNA-1.

	Calc.		Expt.
	Raw^*^	Corrected^**^	
∆*E*(T_1_ → S_1_)	240	180	
∆*E*(T_2_ → S_1_)	−12	−12	
∆*E*(T_1_ → T_2_)	252	192	196
*k*_F_(S_1_ → S_0_)	1.4 × 10^8^ s^−1^	1.4 × 10^8^ s^−1^	1.0 × 10^8^ s^−1^
*k*_IC_(S_1_ → S_0_)	1.2 × 10^7^ s^−1^	1.2 × 10^7^ s^−1^	1.3 × 10^7^ s^−1^
*k*_ISC_(S_1_ → T_1_)	1.7 × 10^5^ s^−1^	2.8 × 10^5^ s^−1^	
*k*_ISC_(S_1_ → T_2_)	5.4 × 10^6^ s^−1^	5.4 × 10^6^ s^−1^	
*k*_IC_(T_2_ →T_1_)	1.8 × 10^11^ s^−1^	5.6 × 10^11^ s^−1^	
*k*_RISC_(T_2_ → S_1_)	2.9 × 10^8^ s^−1^	2.9 × 10^8^ s^−1^	
*k*_Phos_(T_1_ → S_0_)	9.4 × 10^−1^ s^−1^	1.9 × 10^−1^ s^−1^	
*k*_IC_(T_1_ → T_2_)	1.1 × 10^7^ s^−1^	3.3 × 10^8^ s^−1^	
*k*_RISC_(T_1_ → S_1_)	2.5 s^−1^	4.2 × 10^1^ s^−1^	
Φ	0.92	0.92	0.88
Φ_p_	0.89	0.89	0.85
Φ_TADF_	2.9 × 10^−2^	3.2 × 10^−2^	3.5 × 10^−2^
Φ_ISC_	3.5 × 10^−2^	3.5 × 10^−2^	4.0 × 10^−2^
*τ*_TADF_	5.2 × 10^−4^ s	5.1 × 10^−5^ s	9.4 × 10^−5^ s
*k*_TADF_	0.16 × 10^4^ s^−1^	1.8 × 10^4^ s^−1^	0.94 × 10^4^ s^−1^
*k*(T_1_ → T_2_ → S_1_)	0.17 × 10^4^ s^−1^	1.9 × 10^4^ s^−1^	1.0 × 10^4^ s^−1^
*k*(S_1_ → T_2_ → T_1_)	5.4 × 10^6^ s^−1^	5.4 × 10^6^ s^−1^	4.5 × 10^6^ s^−1^

Experimental values are from Hatakeyama et al.^10^.

^*^Energy differences were calculated using the EOM-CCSD/6-31 G method (Fig. 1b,c).

^**^Corrected energy differences were used (Fig. 1d,e).

Calculated values of *k*_F_(S_1_ → S_0_) (1.4 × 10^8 ^s^−1^) and *k*_IC_(S_1_ → S_0_) (1.2 × 10^7 ^s^−1^) agreed quantitatively with experimental results (1.0 × 10^8 ^s^−1^ and 1.3 × 10^7 ^s^−1^, respectively) determined by Hatakeyama et al.^10^ from PL decay curves of a 1-wt% DABNA-1: 9,9′-biphenyl-3,3′-diylbis-9*H*-carbazole film at 300 K (Table 1). The experimentally obtained *k*_ISC_ value (4.5 × 10^6 ^s^−1^) agreed with *k*_ISC_(S_1_ → T_2_) (5.4 × 10^6 ^s^−1^), rather than *k*_ISC_(S_1_ → T_1_) (1.7 × 10^5 ^s^−1^), suggesting that the experimental *k*_ISC_ should be assigned to *k*_ISC_(S_1_ → T_2_). The *k*_ISC_(S_1_ → T_2_) was ten times greater than *k*_ISC_(S_1_ → T_1_), because of the larger S_1_–T_2_ SOC (1.52 cm^−1^) relative to that of S_1_–T_1_ (0.06 cm^−1^). The large S_1_ → T_2_ SOC enhanced S_1_ → T_2_ ISC, despite the uphill transition from S_1_ to T_2_ (the Franck–Condon energy difference was 135 meV, Fig. 1b and Supplementary Table 13), compared with the downhill transition from S_1_ to T_1_ (260 meV). The small S_1_–T_1_ SOC resulted from very similar S_1_ and T_1_ orbital configurations (HOMO → LUMO transition). These results suggested that the ISC (S_1_ → T_1_ conversion) of DABNA-1 occurred via the stepwise S_1_ → T_2_ → T_1_ process, rather than by the one-step S_1_ → T_1_ ISC, irrespective of the geometry relaxation (gray arrows in Fig. 1c). Such T_2_-supported ISC is experimentally observed for a TADF system when ∆*E*(S_1_ → T_2_) is sufficiently small^46^. Because T_2_ → T_1_ IC was much faster than S_1_ → T_2_ ISC, the latter was the rate-determining step of the S_1_ → T_2_ → T_1_ process (Fig. 1c). Hence, the net rate constant *k*(S_1_ → T_2_ → T_1_) was approximately equal to *k*_ISC_ (S_1_ → T_2_) = 5.4 × 10^6 ^s^−1^ (Table 1).

**Fig. 2:**
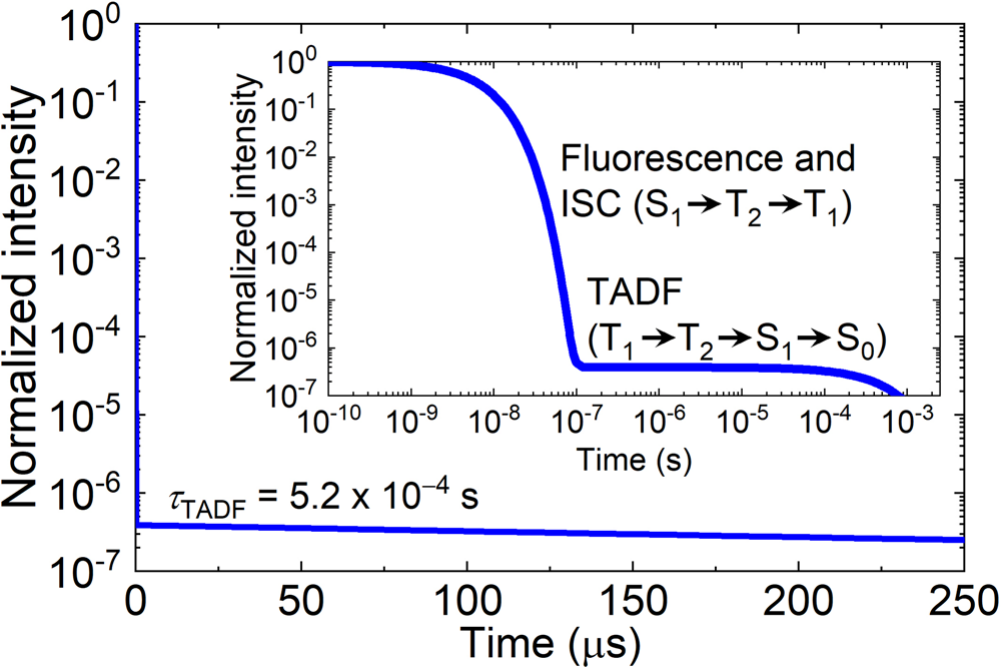
Calculated transient photoluminescence (trPL) decay curve. The inset is a log–log plot of the decay curve.

For RISC from T_1_, the calculated *k*_RISC_(T_1_ → S_1_) (2.5 s^−1^) was considerably less than the experimental value (1 × 10^4 ^s^−1^)^10^, indicating that T_1_ → S_1_ conversion did not occur in one-step but via a more effective process. Because the S_2_ and T_3_ energy levels, as well as higher singlet and triplet states, could not contribute to the RISC from T_1_, as discussed above, and T_2_ was close to S_1_ (see Supplementary Fig. 3), stepwise T_1_ → T_2_ → S_1_ was the most promising mechanism (green arrows in Fig. 1c). To determine *k*(T_1_ → T_2_ → S_1_), we calculated the trPL decay curve (Fig. 2) via kinetic equations (Supplementary Equations S10–S17, Supplementary Table 15, and Supplementary Note 1). The total PLQY (Φ) and the TADF lifetime (*τ*_TADF_) were then obtained from the trPL decay curve (Fig. 2). The prompt and delayed components of Φ (Φ_P_ and Φ_TADF_, respectively) were calculated from

1 $$\begin{array}{lll}{\Phi }_{{{{\rm{P}}}}}=\\ \frac{{k}_{{{{\rm{F}}}}}\left({{{{\rm{S}}}}}_{1}\to {{{{\rm{S}}}}}_{0}\right)}{{k}_{{{{\rm{F}}}}}\left({{{{\rm{S}}}}}_{1}\to {{{{\rm{S}}}}}_{0}\right)+{k}_{{{{\rm{IC}}}}}\left({{{{\rm{S}}}}}_{1}\to {{{{\rm{S}}}}}_{0}\right)+{k}_{{{{\rm{ISC}}}}}\left({{{{\rm{S}}}}}_{1}\to {{{{\rm{T}}}}}_{2}\right)+{k}_{{{{\rm{ISC}}}}}\left({{{{\rm{S}}}}}_{1}\to {{{{\rm{T}}}}}_{1}\right)}\end{array}$$

2 $${\Phi }_{{{{\rm{TADF}}}}}=\Phi -{\Phi }_{{{{\rm{P}}}}}$$

and the ISC quantum yield (Φ_ISC_) was calculated from

3 $$\begin{array}{lll}{\Phi }_{{{{\rm{ISC}}}}}=\\ \frac{{k}_{{{{\rm{ISC}}}}}\left({{{{\rm{S}}}}}_{1}\to {{{{\rm{T}}}}}_{2}\right)+{k}_{{{{\rm{ISC}}}}}\left({{{{\rm{S}}}}}_{1}\to {{{{\rm{T}}}}}_{1}\right)}{{k}_{{{{\rm{F}}}}}\left({{{{\rm{S}}}}}_{1}\to {{{{\rm{S}}}}}_{0}\right)+{k}_{{{{\rm{IC}}}}}\left({{{{\rm{S}}}}}_{1}\to {{{{\rm{S}}}}}_{0}\right)+{k}_{{{{\rm{ISC}}}}}\left({{{{\rm{S}}}}}_{1}\to {{{{\rm{T}}}}}_{2}\right)+{k}_{{{{\rm{ISC}}}}}\left({{{{\rm{S}}}}}_{1}\to {{{{\rm{T}}}}}_{1}\right)}\end{array}$$

Then, *k*_TADF_ was determined from^10^

4 $${k}_{{{{\rm{TADF}}}}}=\frac{{\Phi }_{{{{\rm{TADF}}}}}}{{\Phi }_{{{{\rm{ISC}}}}}{\tau }_{{{{\rm{TADF}}}}}}$$

Finally, *k*(T_1_ → T_2_ → S_1_) was calculated from

5 $$k\left({{{{\rm{T}}}}}_{1}\to {{{{\rm{T}}}}}_{2}\to {{{{\rm{S}}}}}_{1}\right)=\frac{{k}_{{{{\rm{F}}}}}\left({{{{\rm{S}}}}}_{1}\to {{{{\rm{S}}}}}_{0}\right)\times {k}_{{{{\rm{TADF}}}}}}{{k}_{{{{\rm{F}}}}}\left({{{{\rm{S}}}}}_{1}\to {{{{\rm{S}}}}}_{0}\right)-k\left({{{{\rm{S}}}}}_{1}\to {{{{\rm{T}}}}}_{2}\to {{{{\rm{T}}}}}_{1}\right)}$$

Table 1 also lists calculated Φ, Φ_P_, Φ_TADF_, Φ_ISC_, *τ*_TADF_, *k*_TADF_, *k*(T_1_ → T_2_ → S_1_), and *k*(S_1_ → T_2_ → T_1_) values. The Φ, Φ_P_, Φ_TADF_, and Φ_ISC_ values were quantitatively consistent with the experimental values because of the quantitative predictions for *k*_F_(S_1_ → S_0_), *k*_IC_(S_1_ → S_0_), *k*_ISC_(S_1_ → T_2_), and *k*_ISC_(S_1_ → T_1_). The calculated conversion rate constant *k*(S_1_ → T_2_ → T_1_) was consistent with the experimental ${k}_{{{{\rm{ISC}}}}}$, suggesting that the experimental ISC should be assigned to S_1_ → T_2_ → T_1_ ISC rather than direct S_1_ → T_1_ ISC. All the calculated single-step rate constants and *k*(S_1_ → T_2_ → T_1_) agreed well with the experimental values. However, the agreements of the calculated *k*_TADF_ and *k*(T_1_ → T_2_ → S_1_) (0.16 × 10^4 ^s^−1^ and 0.17 × 10^4 ^s^−1^, respectively) and the experiments (0.94 × 10^4 ^s^−1^ and 1.0 × 10^4 ^s^−1^, respectively) were not as good as we expected, compared with the case of *k*(S_1_ → T_2_ → T_1_). Considering that they included the uphill-energy T_1_ → T_2_ IC, there was a possibility that the overestimation of ∆*E*(T_1_ → T_2_) leads to an underestimation of *k*_IC_(T_1_ → T_2_), *k*_TADF_, and *k*(T_1_ → T_2_ → S_1_).

To examine the effect of ∆*E*(T_1_ → T_2_) on *k*_IC_(T_1_ → T_2_), *k*_TADF_, and *k*(T_1_ → T_2_ → S_1_), we recalculated these rate constants using a corrected ∆*E*(T_1_ → T_2_). The rate-determining step for the T_2_-mediated RISC was T_1_ → T_2_ IC; hence, ∆*E*(T_1_ → T_2_) could be viewed as the activation energy for DABNA-1 TADF and RISC. As discussed above, the energy difference between the calculated and experimental T_1_ → S_1_ was 60 meV. Thus, 60 meV was subtracted from the EOM-CCSD-calculated ∆*E*(T_1_ → T_2_) value of 252 meV. The energy levels and rate constants calculated with the corrected ∆*E*(T_1_ → T_2_) are also listed in Table 1. The value of *k*(S_1_ → T_2_ → T_1_) was unchanged, suggesting that the T_1_ energy correction had little effect on the ISC. In contrast, *k*_IC_(T_1_ → T_2_) increased from 1.1 × 10^7 ^s^−1^ to 3.3 × 10^8 ^s^−1^. As a result, *k*_TADF_ and *k*(T_1_ → T_2_ → S_1_) increased to 1.8 × 10^4 ^s^−1^ and 1.9 × 10^4 ^s^−1^, respectively, which were in much better agreements with the experiments. These results suggested that the ∆*E*(T_1_ → T_2_) overestimation was responsible for the underestimation of *k*_TADF_ and *k*(T_1_ → T_2_ → S_1_). Thus, *k*_TADF_ and *k*(T_1_ → T_2_ → S_1_) depended largely on *k*_IC_(T_1_ → T_2_), which was the sum of IC rate constants for individual molecular vibrations, *k*_IC,*α*_(T_1_ → T_2_), where *α* denoted the *α*th vibrational mode (Supplementary Equations S5–S7). A combined in-plane and out-of-plane bending mode (743 cm^−1^) had the largest *k*_IC,*α*_(T_1_ → T_2_) value (Supplementary Table 14 and Supplementary Fig. 5), suggesting that the T_1_ → T_2_ IC was predominantly accelerated by the bending mode.

### Summary

In this work, we calculated the IC, ISC, RISC, fluorescence, phosphorescence, and TADF rate constants for DABNA-1, using EOM-CCSD wave functions and Fermi’s golden rule. The values quantitatively reproduced those from experiments, and thus validated the calculations. Various quantum yields, including PLQY, were also predicted and we revealed the DABNA-1 decay mechanism. The calculated population kinetics and the trPL decay curve indicated that TADF from DABNA-1 occurs via consecutive two processes, T_1_ → T_2_ IC, T_2_ → S_1_ RISC, and S_1_ → S_0_ radiative decay. Our proposed method here will be useful for accurate prediction of all rate constants and quantum yields relevant to OLED phenomena for various compounds with a wide range of structures.

## Methods

### Calculation of matrix elements between vibronic states

Geometry optimization and frequency analysis of S_0_ for DABNA-1 were performed using the TPSSh/6-31+G(d) method, whereas those of S_1_, T_1_, and T_2_ were performed using the TD-TPSSh/6-31+G(d) method with spin multiplicity = 1 (Supplementary Table 1–5 and Supplementary Fig. 1). The polarizable continuum model (PCM) of a CH_2_Cl_2_ solvent was used to consider the effects on vibronic states. We used the functional and the PCM conditions for ground- and excited-state geometry optimizations because Gao et al. reported that the TPSSh/6-31+G(d)-PCM model reproduced experimental DABNA-1 emission and absorption wavelengths in CH_2_Cl_2_^41^. The geometry optimization and frequency analysis were performed with the Gaussian 16 program package^47^. For MR molecules, EOM-CCSD method and algebraic diagrammatic construction of the second order, have been shown to be suitable for calculating singlet–triplet energy differences^42,48–50^. Here, excited-state calculations were performed using the EOM-CCSD/6-31 G method implemented in the Q-Chem program package^51^. Excitation energies, SOCs, vibronic couplings, transition-dipole moments, and permanent dipole moments were calculated using EOM-CCSD wave functions. Examples of Q-Chem input files for running SOC calculations are shown in the Supplementary Methods.

### Calculation of IC rate constant

Mathematical formulation of a method of calculating vibronic couplings and *k*_IC_ is described elsewhere^45,52^ and reviewed in Supplementary Equation S5–S9. First, for each vibrational mode *α* and each ${{{{\rm{S}}}}}_{m}{{\mbox{-}}}{{{{\rm{S}}}}}_{n}$ transition, $\Delta {E}_{{{{\rm{FC}}}}}\left({{{{\rm{S}}}}}_{m}{{\mbox{-}}}{{{{\rm{S}}}}}_{n}\right)$ and ${V}_{\alpha }$ were obtained from the EOM-CCSD calculations (Supplementary Equation S7) and ${{{{\rm{LSF}}}}}_{{{{\rm{IC}}}},\alpha }$ was obtained from the frequency analyses performed using the TPSSh/6-31+G(d)-PCM model (Supplementary Equations S8 and S9). Then, ${k}_{{{{\rm{IC}}}},\alpha }\left({{{{\rm{S}}}}}_{m}{{\mbox{-}}}{{{{\rm{S}}}}}_{n}\right)$ was calculated (Supplementary Equation S6). Finally, ${k}_{{{{\rm{IC}}}},\alpha }\left({{{{\rm{S}}}}}_{m}{{\mbox{-}}}{{{{\rm{S}}}}}_{n}\right)$ was calculated by summing ${k}_{{{{\rm{IC}}}},\alpha }\left({{{{\rm{S}}}}}_{m}{{\mbox{-}}}{{{{\rm{S}}}}}_{n}\right)$ over *α* (Supplementary Equation S5). This protocol was also used for calculating the IC rate constants for ${{{{\rm{T}}}}}_{m}{{\mbox{-}}}{{{{\rm{T}}}}}_{n}$ transitions.

## Supplementary information

Supplementary Information

Peer Review File

## Data Availability

All relevant data are available from the authors upon request.

## Code availability

The code used to generate Fig. 2 is available from the authors upon request.

## References

[1] Uoyama H, Goushi K, Shizu K, Nomura H, Adachi C (2012). Highly efficient organic light-emitting diodes from delayed fluorescence. Nature.

[2] Adachi C (2014). Third-generation organic electroluminescence materials. Jpn. J. Appl. Phys..

[3] Wong MY, Zysman-Colman E (2017). Purely organic thermally activated delayed fluorescence materials for organic light-emitting diodes. Adv. Mater..

[4] Tanaka H, Shizu K, Miyazaki H, Adachi C (2012). Efficient green thermally activated delayed fluorescence (TADF) from a phenoxazine-triphenyltriazine (PXZ-TRZ) derivative. Chem. Commun..

[5] Zhang Q (2012). Design of efficient thermally activated delayed fluorescence materials for pure blue organic light emitting diodes. J. Am. Chem. Soc..

[6] Kaji H (2015). Purely organic electroluminescent material realizing 100% conversion from electricity to light. Nat. Commun..

[7] Shizu K (2015). Strategy for designing electron donors for thermally activated delayed fluorescence emitters. J. Phys. Chem. C.

[8] Shizu K (2015). Enhanced electroluminescence from a thermally activated delayed-fluorescence emitter by suppressing nonradiative decay. Phys. Rev. Appl..

[9] Wada Y, Nakagawa H, Matsumoto S, Wakisaka Y, Kaji H (2020). Organic light emitters exhibiting very fast reverse intersystem crossing. Nat. Photon..

[10] Hatakeyama T (2016). Ultrapure blue thermally activated delayed fluorescence molecules: efficient HOMO–LUMO separation by the multiple resonance effect. Adv. Mater..

[11] Nakatsuka S, Gotoh H, Kinoshita K, Yasuda N, Hatakeyama T (2017). Divergent synthesis of heteroatom-centered 4,8,12-triazatriangulenes. Angew. Chem. Int. Ed..

[12] Matsui K (2018). One-shot multiple borylation toward BN-doped nanographenes. J. Am. Chem. Soc..

[13] Liang X (2018). Peripheral amplification of multi-resonance induced thermally activated delayed fluorescence for highly efficient OLEDs. Angew. Chem. Int. Ed..

[14] Han SH, Jeong JH, Yoo JW, Lee JY (2019). Ideal blue thermally activated delayed fluorescence emission assisted by a thermally activated delayed fluorescence assistant dopant through a fast reverse intersystem crossing mediated cascade energy transfer process. J. Mater. Chem. C.

[15] Kondo Y (2019). Narrowband deep-blue organic light-emitting diode featuring an organoboron-based emitter. Nat. Photon..

[16] Oda S, Kawakami B, Kawasumi R, Okita R, Hatakeyama T (2019). Multiple resonance effect-induced sky-blue thermally activated delayed fluorescence with a narrow emission band. Org. Lett..

[17] Zhang Y (2019). Multi-resonance induced thermally activated delayed fluorophores for narrowband green OLEDs. Angew. Chem. Int. Ed..

[18] Suresh SM (2020). A deep blue B,N-doped heptacene emitter that shows both thermally activated delayed fluorescence and delayed fluorescence by triplet–triplet annihilation. J. Am. Chem. Soc..

[19] Zhang Y (2020). Achieving pure green electroluminescence with CIEy of 0.69 and EQE of 28.2% from an Aza-fused multi-resonance emitter. Angew. Chem. Int. Ed..

[20] Yang M, Park IS, Yasuda T (2020). Full-color, narrowband, and high-efficiency electroluminescence from boron and carbazole embedded polycyclic heteroaromatics. J. Am. Chem. Soc..

[21] Madayanad Suresh S, Hall D, Beljonne D, Olivier Y, Zysman-Colman E (2020). Multiresonant thermally activated delayed fluorescence emitters based on heteroatom-doped nanographenes: recent advances and prospects for organic light-emitting diodes. Adv. Funct. Mater..

[22] Tanaka H (2021). Hypsochromic shift of multiple-resonance-induced thermally activated delayed fluorescence by oxygen atom incorporation. Angew. Chem. Int. Ed..

[23] Oda S (2021). Carbazole-based DABNA analogues as highly efficient thermally activated delayed fluorescence materials for narrowband organic light-emitting diodes. Angew. Chem. Int. Ed..

[24] Nagata M (2021). Fused-nonacyclic multi-resonance delayed fluorescence emitter based on ladder-thiaborin exhibiting narrowband sky-blue emission with accelerated reverse intersystem crossing. Angew. Chem. Int. Ed..

[25] Yang M (2021). Wide-range color tuning of narrowband emission in multi-resonance organoboron delayed fluorescence materials through rational imine/amine functionalization. Angew. Chem. Int. Ed..

[26] Yuan Y (2019). The design of fused amine/carbonyl system for efficient thermally activated delayed fluorescence: novel multiple resonance core and electron acceptor. Adv. Opt. Mater..

[27] Hall D (2020). Improving processability and efficiency of resonant TADF emitters: a design strategy. Adv. Opt. Mater..

[28] Naveen, K. R. et al. Achieving high efficiency and pure blue color in hyperfluorescence organic light emitting diodes using organo-boron based emitters. *Adv. Funct. Mater*. **32**, 2110356 (2022).

[29] Ikeda N (2020). Solution-processable pure green thermally activated delayed fluorescence emitter based on the multiple resonance effect. Adv. Mater..

[30] Sun D (2020). The design of an extended multiple resonance TADF emitter based on a polycyclic amine/carbonyl system. Mater. Chem. Front..

[31] Liu G (2021). Facile synthesis of multi-resonance ultra-pure-green TADF emitters based on bridged diarylamine derivatives for efficient OLEDs with narrow emission. J. Mater. Chem. C.

[32] Hua T (2021). Heavy-atom effect promotes multi-resonance thermally activated delayed fluorescence. Chem. Eng. J..

[33] Jiang P (2021). Simple acridan-based multi-resonance structures enable highly efficient narrowband green TADF electroluminescence. Adv. Opt. Mater..

[34] Jiang P (2021). Quenching-resistant multiresonance TADF emitter realizes 40% external quantum efficiency in narrowband electroluminescence at high doping level. Adv. Mater..

[35] Wu X (2022). Fabrication of circularly polarized MR-TADF emitters with asymmetrical peripheral-lock enhancing helical B/N-doped nanographenes. Adv. Mater..

[36] Xu Y, Wang Q, Cai X, Li C, Wang Y (2021). Highly efficient electroluminescence from narrowband green circularly polarized multiple resonance thermally activated delayed fluorescence enantiomers. Adv. Mater..

[37] Xu Y (2021). Materials with high color purity based on strong acceptor attachment onto B–N-containing multiple resonance frameworks. CCS Chem..

[38] Liu Y, Xiao X, Ran Y, Bin Z, You J (2021). Molecular design of thermally activated delayed fluorescent emitters for narrowband orange–red OLEDs boosted by a cyano-functionalization strategy. Chem. Sci..

[39] Zhang Y (2021). Multi-resonance deep-red emitters with shallow potential-energy surfaces to surpass energy-gap law. Angew. Chem. Int. Ed..

[40] Northey T, Penfold TJ (2018). The intersystem crossing mechanism of an ultrapure blue organoboron emitter. Org. Electron..

[41] Gao Y (2018). Realizing performance improvement of blue thermally activated delayed fluorescence molecule DABNA by introducing substituents on the para-position of boron atom. Chem. Phys. Lett..

[42] Pershin A (2019). Highly emissive excitons with reduced exchange energy in thermally activated delayed fluorescent molecules. Nat. Commun..

[43] Hall, D. et al. The modelling of multi-resonant thermally activated delayed fluorescence emitters – properly accounting for electron correlation is key! *Preprint at* https://chemrxiv.org/engage/chemrxiv/article-details/6173bad70c048095df40e5f3 (2021).

[44] Lin L, Fan J, Cai L, Wang C-K (2018). Excited state dynamics of new-type thermally activated delayed fluorescence emitters: theoretical view of light-emitting mechanism. Mol. Phys..

[45] Shizu K, Kaji H (2021). Theoretical determination of rate constants from excited states: application to benzophenone. J. Phys. Chem. A.

[46] Han C (2021). Ladder-like energy-relaying exciplex enables 100% internal quantum efficiency of white TADF-based diodes in a single emissive layer. Nat. Commun..

[47] Frisch, M. J. et al. Gaussian 16 Rev. C.01. (2016).

[48] de Silva P (2019). Inverted singlet–triplet gaps and their relevance to thermally activated delayed fluorescence. J. Phys. Chem. Lett..

[49] Pios S, Huang X, Sobolewski AL, Domcke W (2021). Triangular boron carbon nitrides: an unexplored family of chromophores with unique properties for photocatalysis and optoelectronics. Phys. Chem. Chem. Phys..

[50] Ehrmaier J (2019). Singlet–triplet inversion in heptazine and in polymeric carbon nitrides. J. Phys. Chem. A.

[51] Shao Y (2015). Advances in molecular quantum chemistry contained in the Q-Chem 4 program package. Mol. Phys..

[52] Shizu K, Adachi C, Kaji H (2020). Effect of vibronic coupling on correlated triplet pair formation in the singlet fission process of linked tetracene dimers. J. Phys. Chem. A.

