# Supplementary material for: Comprehensive understanding of multiple resonance thermally activated delayed fluorescence through quantum chemistry calculations
Source: Commun Chem. 2022 Apr 14;5:53. doi: 10.1038/s42004-022-00668-6 (PMC9814892; doi:10.1038/s42004-022-00668-6)
Supplement: Supplementary file 1 — Supplementary Information [file 42004_2022_668_MOESM1_ESM.pdf]

Supplementary Information for

Comprehensive understanding of multiple resonance thermally activated delayed fluorescence through quantum chemistry calculations

*Katsuyuki Shizu[1] and Hironori Kaji[1]**

Email: kaji@scl.kyoto-u.ac.jp

[1]Institute for Chemical Research, Kyoto University, Uji, Kyoto 611-0011, Japan

Supplementary methods

Full geometry optimization and frequency analysis of S_0 of DABNA-1

The geometry of ground state, S_0, of DABNA-1 was optimized at the TPSSh/6-31+G(d) level of theory using the polarizable continuum model (PCM, CH_2Cl_2) and then, its stability was examined using frequency analysis at the same level of theory. No imaginary modes were obtained, indicating that the optimized S_0 geometry was stationary minima. The geometry optimization and frequency analysis were performed using the Gaussian 16 package.[1] Supplementary Table 1 lists the standard nuclear orientation.

Full geometry optimization and frequency analysis of S_1 of DABNA-1

The geometry of S_1 of DABNA-1 was optimized at the TD-TPSSh/6-31+G(d) level of theory using the polarizable continuum model (PCM, CH_2Cl_2) and then, its stability was examined using frequency analysis at the same level of theory. No imaginary modes were obtained, indicating that the optimized S_1 geometry was stationary minima. The geometry optimization and frequency analysis were performed using the Gaussian 16 package.[1] Supplementary Table 2 lists the standard nuclear orientation.

Full geometry optimization and frequency analysis of T_1 of DABNA-1

The geometry of T_1 of DABNA-1 was optimized at the TD-TPSSh/6-31+G(d) level of theory using the polarizable continuum model (PCM, CH_2Cl_2) and then, its stability was examined using frequency analysis at the same level of theory. No imaginary modes were obtained, indicating that the optimized T_1 geometry was stationary minima. The geometry optimization and frequency analysis were performed using the Gaussian 16 package.[1] Supplementary Table 3 lists the standard nuclear orientation.

Full geometry optimization and frequency analysis of T_2 of DABNA-1

The geometry of T_2 of DABNA-1 was optimized at the TD-TPSSh/6-31G+(d) level of theory using the polarizable continuum model (PCM, CH_2Cl_2) and then, its stability was examined using frequency analysis at the same level of theory. No imaginary modes were obtained, indicating that the optimized T_2 geometry was stationary minima. The geometry optimization and frequency analysis were performed using the Gaussian 16 package.[1] Supplementary Table 4 lists the standard nuclear orientation.

Calculation of spin-orbit couplings

Spin-orbit couplings were calculated at the EOM-CCSD/6-31G level of theory using the Q-Chem program package.[2] For S_1-T_1, S_1-T_2, S_1-T_3, and S_1-T_4 SOCs, we added the following key words in a Q-Chem input file.

```
$rem
   METHOD          eom-ccsd
   BASIS           6-31G
   EE_SINGLETS     [2,2]
   EE_TRIPLETS     [2,2]
   GUI             2
   Mem_Static      512000
   Mem_Total       1000000
   ao2mo_disk      1000000
   CC_TRANS_PROP     = true
   CALC_SOC          = true
   CC_STATE_TO_OPT = [2,1]
$end
```

For S_0-T_1, S_0-T_2, S_0-T_3, and S_0-T_4 SOCs, we added the following key words in a Q-Chem input file.

```
$rem
   METHOD          eom-ccsd
   BASIS           6-31G
   EE_SINGLETS     [2,2]
   EE_TRIPLETS     [2,2]
   GUI             2
   Mem_Static      512000
   Mem_Total       1000000
   ao2mo_disk      1000000
   CC_TRANS_PROP = true
   CALC_SOC      = true
$end
```

Mathematical formulae for rate constants

The rate constant for $S_1 \rightarrow S_0$ fluorescence ($k_F(S_1 \rightarrow S_0)$) can be written in terms of the S_1-S_0 adiabatic energy difference ($\Delta E_{AD}(S_1\text{-}S_0)$), S_1-S_0 transition dipole moment ($\mu_F(S_1\text{-}S_0)$), the vacuum electric permittivity (ε_0), the Dirac constant ($\hbar$), and the speed of light (c):

$$k_{\mathrm{F}} = \frac{4\Delta E_{\mathrm{AD}}(\mathrm{S}_1 - \mathrm{S}_0)^3}{3\epsilon_0 \hbar^4 c^3} \mu_{\mathrm{F}}(\mathrm{S}_1 - \mathrm{S}_0)^2 \quad \text{(S1)}$$

The rate constant for $T_n \rightarrow S_0$ ($n \geq 1$) phosphorescence ($k_{Phos}(T_n\text{-}S_0)$) can be written in terms of the adiabatic T_n-S_0 energy difference ($\Delta E_{AD}(T_n\text{-}S_0)$) and T_n-S_0 transition dipole moment ($\mu_{Phos}(T_n\text{-}S_0)$):

$$k_{\mathrm{Phos}}(\mathrm{T}_n\text{-}\mathrm{S}_0) = \frac{4\Delta E_{\mathrm{AD}}(\mathrm{T}_n\text{-}\mathrm{S}_0)^3}{3\epsilon_0 \hbar^4 c^3} \mu_{\mathrm{Phos}}(\mathrm{T}_n\text{-}\mathrm{S}_0)^2 \quad \text{(S2)}$$

The rate constant for S_m-T_n ($m \geq 0$, $n \geq 1$) intersystem crossing ($k_{ISC}(S_m\text{-}T_n)$) can be written in terms of the adiabatic S_m-T_n energy difference $\Delta E_{AD}(S_m\text{-}T_n)$, the norm of the S_m-T_n spin-orbit coupling ($|\mathrm{SOC}(S_m\text{-}T_n)|$), and line shape function ($\mathrm{LSF_{ISC}}$):

$$k_{\mathrm{ISC}}(\mathrm{S}_m\text{-}\mathrm{T}_n) = \frac{2\pi}{\hbar} |\mathrm{SOC}(\mathrm{S}_m\text{-}\mathrm{T}_n)|^2 \times \mathrm{LSF_{ISC}}\left(\Delta E_{\mathrm{AD}}(\mathrm{S}_m\text{-}\mathrm{T}_n)\right) \quad \text{(S3)}$$

$$\mathrm{LSF_{ISC}}\left(\Delta E_{\mathrm{AD}}(\mathrm{S}_m\text{-}\mathrm{T}_n)\right) = \frac{1}{\pi} \frac{\gamma}{\Delta E_{\mathrm{AD}}(\mathrm{S}_m\text{-}\mathrm{T}_n)^2 + \gamma^2} \quad \text{(S4)}$$

where γ is the broadening of $\mathrm{LSF_{ISC}}$. γ was set to be 600 cm^{-1} (see Supplementary Figure 4). When the $S_m \rightarrow T_n$ transition is an energy-downhill transition, the energy-downhill $S_m \rightarrow T_n$ ISC rate constant is expresses as $k_{ISC}(S_m\text{-}T_n)$, whereas the energy-uphill $T_n \rightarrow S_m$ rate constant is calculated as $k_{ISC}(S_m\text{-}T_n)\exp\{-\beta \Delta E_{FC}(S_m\text{-}T_n)\}/3$, where β is the inverse temperature ($\beta^{-1} = k_B T$, where k_B is the Boltzmann constant and T is the temperature). When the $S_m \rightarrow T_n$ transition is an energy-uphill transition, the energy-downhill $T_n \rightarrow S_m$ ISC rate constant is expresses as $k_{ISC}(S_m\text{-}T_n)/3$, whereas the energy-uphill S_m-T_n rate constant is calculated as $k_{ISC}(S_m\text{-}T_n)\exp\{-\beta \Delta E_{FC}(S_m\text{-}T_n)\}$.

The rate constant for S_m-S_n ($m > n \geq 0$, $m \neq n$) internal conversion ($k_{IC}(S_m\text{-}S_n)$) can be written in terms of the Franck-Condon S_m-S_n energy difference $\Delta E_{FC}(S_m\text{-}S_n)$, the adiabatic S_m-S_n energy difference $\Delta E_{AD}(S_m\text{-}S_n)$, the S_m-S_n vibronic coupling ($V_\alpha(S_m\text{-}S_n)$), and line shape function ($\mathrm{LSF_{IC}}$):

$$k_{\mathrm{IC}}(\mathrm{S}_m\text{-}\mathrm{S}_n) = \sum_\alpha k_{\mathrm{IC},\alpha}(\mathrm{S}_m\text{-}\mathrm{S}_n) \quad \text{(S5)}$$

$$k_{\mathrm{IC},\alpha}(\mathrm{S}_m\text{-}\mathrm{S}_n) = \frac{R_\alpha}{\hbar} \mathrm{LSF}_{\mathrm{IC},\alpha} \quad \text{(S6)}$$

$$R_\alpha = \frac{\hbar^2 {V_\alpha}^2}{\Delta E_{\mathrm{FC}}(\mathrm{S}_m\text{-}\mathrm{S}_n)^2} \quad \text{(S7)}$$

$$\mathrm{LSF}_{\mathrm{IC},\alpha} = 2\pi\sinh\left(\frac{1}{2}\beta\hbar\omega_\alpha\right)\sum_{v_\alpha}\exp\left\{-\left(\frac{1}{2}+v_\alpha\right)\beta\hbar\omega_\alpha\right\}$$

$$\times\frac{1}{\pi S}\left\{\frac{v_\alpha\gamma}{\{\Delta E_{\mathrm{AD}}(\mathrm{S}_m\text{-}\mathrm{S}_n)+\hbar\omega_\alpha\}^2+\gamma^2}+\frac{(v_\alpha+1)\gamma}{\{\Delta E_{\mathrm{AD}}(\mathrm{S}_m\text{-}\mathrm{S}_n)-\hbar\omega_\alpha\}^2+\gamma^2}\right\} \tag{S8}$$

$$S=\frac{1}{4\pi\epsilon_0}\frac{e^2}{a_B hc} \tag{S9}$$

where v_α and ω_α are the vibrational quantum number and angular frequency for the αth vibrational mode, respectively, e is the elementary charge, a_{B} is the Bohr radius, and h is the Planck constant. The energy-downhill $\mathrm{S}_m \rightarrow \mathrm{S}_n$ rate constant is calculated as $k_{\mathrm{IC}}(\mathrm{S}_m\text{-}\mathrm{S}_n)$, whereas the energy-uphill $\mathrm{S}_n \rightarrow \mathrm{S}_m$ rate constant is calculated as $k_{\mathrm{IC}}(\mathrm{S}_m\text{-}\mathrm{S}_n)\exp\{-\beta\Delta E_{\mathrm{FC}}(\mathrm{S}_m\text{-}\mathrm{S}_n)\}$.

The rate constant for $\mathrm{T}_m \rightarrow \mathrm{T}_n$ ($m > n \geq 1$, $m \neq n$) internal conversion ($k_{\mathrm{IC}}(\mathrm{T}_m \rightarrow \mathrm{T}_n)$) can be calculated in a way similar to that for $k_{\mathrm{IC}}(\mathrm{S}_m\text{-}\mathrm{S}_n)$.

Supplementary Table 1. Standard nuclear orientation of S_0 geometry of DABNA-1 optimized at the TPSSh/6-31+G(d) level of theory (PCM: dichloromethane).

Atom	Element	x (Å)	y (Å)	z (Å)
1	C	0.535385	-3.982201	-3.098962
2	C	0.291123	-3.836493	-1.739293
3	C	0.167113	-2.547190	-1.165355
4	C	-0.224781	1.383157	-1.991430
5	C	0.516824	-1.591686	-3.362905
6	C	0.676432	-2.854038	-3.922739
7	N	0.000000	2.440606	0.225242
8	C	0.002131	1.224897	0.922006
9	C	0.000000	0.000000	0.190145
10	B	0.000000	0.000000	-1.328485
11	C	-0.002131	-1.224897	0.922006
12	N	0.000000	-2.440606	0.225242
13	C	-0.167113	2.547190	-1.165355
14	C	0.224781	-1.383157	-1.991430
15	C	-0.291123	3.836493	-1.739293
16	C	-0.535385	3.982201	-3.098962
17	C	-0.676432	2.854038	-3.922739
18	C	-0.516824	1.591686	-3.362905
19	C	0.016266	1.221955	2.330130
20	C	0.000000	0.000000	3.001993
21	C	-0.016266	-1.221955	2.330130
22	C	0.098543	3.657149	0.999736
23	C	-0.098543	-3.657149	0.999736
24	C	-1.061915	4.277118	1.476075
25	C	-0.958171	5.452664	2.226917
26	C	0.300090	6.003261	2.499744
27	C	1.456862	5.377048	2.020697
28	C	1.358434	4.200872	1.269176
29	C	1.061915	-4.277118	1.476075
30	C	0.958171	-5.452664	2.226917
31	C	-0.300090	-6.003261	2.499744
32	C	-1.456862	-5.377048	2.020697
33	C	-1.358434	-4.200872	1.269176
34	H	0.635973	-4.981949	-3.514150
35	H	0.214001	-4.721567	-1.118244
36	H	0.647283	-0.725438	-4.002707
37	H	0.905128	-2.965802	-4.979151
38	H	-0.214001	4.721567	-1.118244
39	H	-0.635973	4.981949	-3.514150
40	H	-0.905128	2.965802	-4.979151
41	H	-0.647283	0.725438	-4.002707

42	H	0.026773	2.146161	2.895102
43	H	0.000000	0.000000	4.089274
44	H	-0.026773	-2.146161	2.895102
45	H	-2.032007	3.839418	1.257418
46	H	-1.857920	5.936264	2.597296
47	H	0.378440	6.916595	3.083057
48	H	2.434726	5.801708	2.230498
49	H	2.247945	3.704286	0.891554
50	H	2.032007	-3.839418	1.257418
51	H	1.857920	-5.936264	2.597296
52	H	-0.378440	-6.916595	3.083057
53	H	-2.434726	-5.801708	2.230498
54	H	-2.247945	-3.704286	0.891554

Supplementary Table 2. Standard nuclear orientation of S_1 geometry of DABNA-1 optimized at the TD-TPSSh/6-31+G(d) level of theory (PCM: dichloromethane).

Atom	Element	x (Å)	y (Å)	z (Å)
1	C	0.348176	-4.054431	-3.103502
2	C	0.189590	-3.858486	-1.721211
3	C	0.113186	-2.559776	-1.186544
4	C	-0.152932	1.390306	-2.025884
5	C	0.355077	-1.652816	-3.411572
6	C	0.448567	-2.942487	-3.944181
7	N	0.000000	2.423100	0.224458
8	C	0.005112	1.205271	0.897972
9	C	0.000000	0.000000	0.164056
10	B	0.000000	0.000000	-1.376141
11	C	-0.005112	-1.205271	0.897972
12	N	0.000000	-2.423100	0.224458
13	C	-0.113186	2.559776	-1.186544
14	C	0.152932	-1.390306	-2.025884
15	C	-0.189590	3.858486	-1.721211
16	C	-0.348176	4.054431	-3.103502
17	C	-0.448567	2.942487	-3.944181
18	C	-0.355077	1.652816	-3.411572
19	C	0.014664	1.208646	2.326555
20	C	0.000000	0.000000	3.019239
21	C	-0.014664	-1.208646	2.326555
22	C	0.057727	3.636435	1.012495
23	C	-0.057727	-3.636435	1.012495
24	C	-1.127721	4.234414	1.452668
25	C	-1.063530	5.401485	2.221041
26	C	0.177652	5.965114	2.541196
27	C	1.358120	5.361294	2.092590
28	C	1.301818	4.193467	1.323783
29	C	1.127721	-4.234414	1.452668
30	C	1.063530	-5.401485	2.221041
31	C	-0.177652	-5.965114	2.541196
32	C	-1.358120	-5.361294	2.092590
33	C	-1.301818	-4.193467	1.323783
34	H	0.403388	-5.064520	-3.499712
35	H	0.134707	-4.724030	-1.071486
36	H	0.464601	-0.814750	-4.089956
37	H	0.597612	-3.075006	-5.013583
38	H	-0.134707	4.724030	-1.071486
39	H	-0.403388	5.064520	-3.499712
40	H	-0.597612	3.075006	-5.013583
41	H	-0.464601	0.814750	-4.089956

42	H	0.029006	2.141104	2.876925
43	H	0.000000	0.000000	4.104379
44	H	-0.029006	-2.141104	2.876925
45	H	-2.083462	3.786415	1.195837
46	H	-1.981362	5.866815	2.569373
47	H	0.224381	6.871268	3.138699
48	H	2.322668	5.795385	2.340530
49	H	2.209401	3.714125	0.968024
50	H	2.083462	-3.786415	1.195837
51	H	1.981362	-5.866815	2.569373
52	H	-0.224381	-6.871268	3.138699
53	H	-2.322668	-5.795385	2.340530
54	H	-2.209401	-3.714125	0.968024

Supplementary Table 3. Standard nuclear orientation of T_1 geometry of DABNA-1 optimized at the TD-TPSSh/6-31+G(d) level of theory (PCM: dichloromethane).

Atom	Element	x (Å)	y (Å)	z (Å)
1	C	0.353545	-4.056793	-3.102006
2	C	0.193557	-3.862969	-1.723284
3	C	0.113124	-2.563171	-1.184438
4	C	-0.150451	1.392265	-2.023385
5	C	0.353082	-1.653749	-3.406397
6	C	0.451389	-2.942208	-3.940471
7	N	0.000000	2.431273	0.220845
8	C	0.002072	1.206838	0.901081
9	C	0.000000	0.000000	0.154521
10	B	0.000000	0.000000	-1.368657
11	C	-0.002072	-1.206838	0.901081
12	N	0.000000	-2.431273	0.220845
13	C	-0.113124	2.563171	-1.184438
14	C	0.150451	-1.392265	-2.023385
15	C	-0.193557	3.862969	-1.723284
16	C	-0.353545	4.056793	-3.102006
17	C	-0.451389	2.942208	-3.940471
18	C	-0.353082	1.653749	-3.406397
19	C	0.009147	1.208648	2.319466
20	C	0.000000	0.000000	3.020114
21	C	-0.009147	-1.208648	2.319466
22	C	0.065902	3.642797	1.011325
23	C	-0.065902	-3.642797	1.011325
24	C	-1.114770	4.245264	1.457714
25	C	-1.042001	5.412309	2.225025
26	C	0.202931	5.970267	2.540060
27	C	1.378698	5.360261	2.087399
28	C	1.313819	4.192475	1.319721
29	C	1.114770	-4.245264	1.457714
30	C	1.042001	-5.412309	2.225025
31	C	-0.202931	-5.970267	2.540060
32	C	-1.378698	-5.360261	2.087399
33	C	-1.313819	-4.192475	1.319721
34	H	0.412620	-5.065755	-3.500169
35	H	0.140653	-4.728665	-1.073540
36	H	0.458316	-0.815944	-4.085662
37	H	0.601962	-3.072424	-5.009860
38	H	-0.140653	4.728665	-1.073540
39	H	-0.412620	5.065755	-3.500169
40	H	-0.601962	3.072424	-5.009860
41	H	-0.458316	0.815944	-4.085662

42	H	0.019271	2.142253	2.869145
43	H	0.000000	0.000000	4.104897
44	H	-0.019271	-2.142253	2.869145
45	H	-2.073721	3.802047	1.204757
46	H	-1.956306	5.882706	2.575743
47	H	0.256293	6.876861	3.136296
48	H	2.346251	5.790137	2.330831
49	H	2.217783	3.708615	0.960968
50	H	2.073721	-3.802047	1.204757
51	H	1.956306	-5.882706	2.575743
52	H	-0.256293	-6.876861	3.136296
53	H	-2.346251	-5.790137	2.330831
54	H	-2.217783	-3.708615	0.960968

Supplementary Table 4. Standard nuclear orientation of T_2 geometry of DABNA-1 optimized at the TD-TPSSh/6-31+G(d) level of theory (PCM: dichloromethane).

Atom	Element	x (Å)	y (Å)	z (Å)
1	C	0.432319	-4.024599	-3.127583
2	C	0.225134	-3.846008	-1.737094
3	C	0.141616	-2.544298	-1.175309
4	C	-0.200227	1.380404	-2.002691
5	C	0.457614	-1.616633	-3.382798
6	C	0.567655	-2.915017	-3.944782
7	N	0.000000	2.434548	0.217122
8	C	0.000163	1.218501	0.935275
9	C	0.000000	0.000000	0.194704
10	B	0.000000	0.000000	-1.344383
11	C	-0.000163	-1.218501	0.935275
12	N	0.000000	-2.434548	0.217122
13	C	-0.141616	2.544298	-1.175309
14	C	0.200227	-1.380404	-2.002691
15	C	-0.225134	3.846008	-1.737094
16	C	-0.432319	4.024599	-3.127583
17	C	-0.567655	2.915017	-3.944782
18	C	-0.457614	1.616633	-3.382798
19	C	0.005143	1.220688	2.342321
20	C	0.000000	0.000000	3.023051
21	C	-0.005143	-1.220688	2.342321
22	C	0.071632	3.649948	0.997756
23	C	-0.071632	-3.649948	0.997756
24	C	-1.105516	4.260899	1.442558
25	C	-1.026885	5.430486	2.205616
26	C	0.221030	5.980677	2.522613
27	C	1.393598	5.360486	2.075479
28	C	1.322315	4.190336	1.311691
29	C	1.105516	-4.260899	1.442558
30	C	1.026885	-5.430486	2.205616
31	C	-0.221030	-5.980677	2.522613
32	C	-1.393598	-5.360486	2.075479
33	C	-1.322315	-4.190336	1.311691
34	H	0.492570	-5.032338	-3.528628
35	H	0.131041	-4.721017	-1.105074
36	H	0.612066	-0.765707	-4.037427
37	H	0.754744	-3.027513	-5.009693
38	H	-0.131041	4.721017	-1.105074
39	H	-0.492570	5.032338	-3.528628
40	H	-0.754744	3.027513	-5.009693
41	H	-0.612066	0.765707	-4.037427

42	H	0.007289	2.147943	2.902601
43	H	0.000000	0.000000	4.109688
44	H	-0.007289	-2.147943	2.902601
45	H	-2.066928	3.822691	1.190174
46	H	-1.938914	5.909772	2.550401
47	H	0.279152	6.889420	3.115211
48	H	2.363502	5.785512	2.318386
49	H	2.223930	3.697845	0.958736
50	H	2.066928	-3.822691	1.190174
51	H	1.938914	-5.909772	2.550401
52	H	-0.279152	-6.889420	3.115211
53	H	-2.363502	-5.785512	2.318386
54	H	-2.223930	-3.697845	0.958736

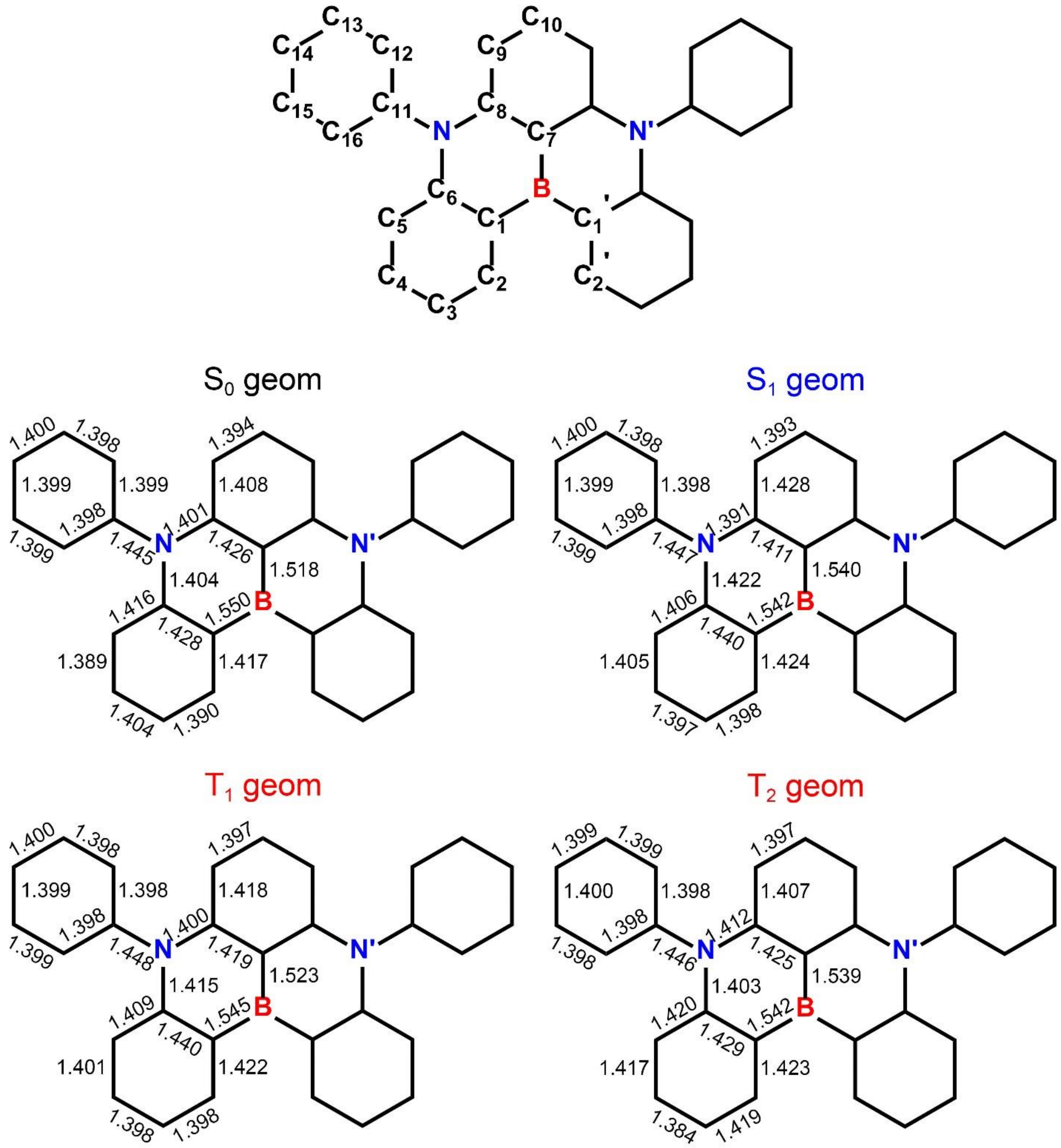

Supplementary Figure 1. Atom labelling scheme and bond lengths (Å) of DABNA-1.

Supplementary Table 5. Calculated bond lengths (Å) and dihedral angles (degree) of DABNA-1. The atom labelling scheme is shown in Supplementary Figure 1.

	S_0 geom	S_1 geom	T_1 geom	T_2 geom
Bond lengths				
C_1–C_2	1.42	1.42	1.42	1.42
C_2–C_3	1.39	1.40	1.40	1.42
C_3–C_4	1.40	1.40	1.40	1.38
C_4–C_5	1.39	1.41	1.40	1.42
C_5–C_6	1.42	1.41	1.41	1.42
C_6–C_1	1.43	1.44	1.44	1.43
B–C_1	1.55	1.54	1.55	1.54
B–C_7	1.52	1.54	1.52	1.54
N–C_6	1.40	1.42	1.42	1.40
N–C_{11}	1.45	1.45	1.45	1.45
N–C_8	1.40	1.39	1.40	1.41
C_8–C_7	1.43	1.41	1.42	1.43
C_8–C_9	1.41	1.43	1.42	1.41
C_9–C_{10}	1.39	1.39	1.40	1.40
C_{11}–C_{12}	1.40	1.40	1.40	1.40
C_{12}–C_{13}	1.40	1.40	1.40	1.40
C_{13}–C_{14}	1.40	1.40	1.40	1.40
C_{14}–C_{15}	1.40	1.40	1.40	1.40
C_{15}–C_{16}	1.40	1.40	1.40	1.40
C_{16}–C_{11}	1.40	1.40	1.40	1.40
Dihedral angles				
B–C_1–C_1'–C_7	0.00	0.00	0.00	0.00
C_2–C_1– C_1'– C_2'	20.1	14.3	14.3	18.2
C_6–N– C_{11}– C_{12}	87.6	88.1	88.3	87.0

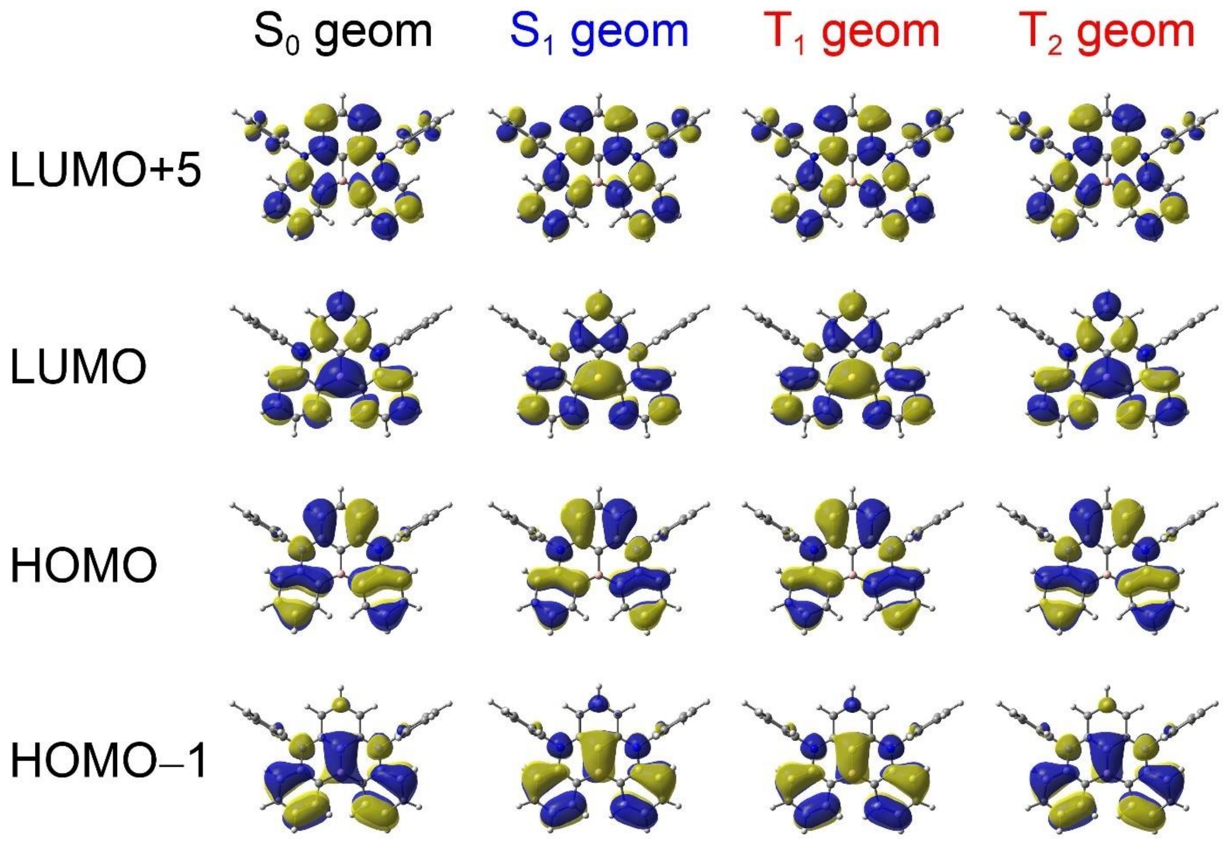

Supplementary Figure 2. LUMO+5, LUMO, HOMO, and HOMO−1 of DABNA-1 calculated for the S_0, S_1, T_1, and T_2 geometries. The molecular orbitals were calculated at the RHF/6-31G level of theory. The S_0 geometry was optimized at the TPSSh/6-31+G(d) level of theory (PCM: dichloromethane); the S_1, T_1, and T_2 geometries were optimized at the TD-TPSSh/6-31+G(d) level of theory (PCM: dichloromethane).

Supplementary Table 6. S_0, S_1, T_1, and T_2 of DABNA-1 calculated at the EOM-CCSD/6-31G level of theory. The optimized S_0 geometry in Supplementary Table 1 was used. Φ_0 denotes the Hartree-Fock determinant. Φ_a^r and Φ_{ab}^{rs} denote single and double excitations from Φ_0, respectively.

State	Excitation energy (eV)	Slater determinant	Contribution (%)
S_0		Φ_0	98.18
		$\Phi_{\mathrm{HOMO-1}}^{\mathrm{LUMO}}$	0.28
		$\Phi_{\overline{\mathrm{HOMO-1}}}^{\overline{\mathrm{LUMO}}}$	0.28
		$\Phi_{\mathrm{HOMO-5}\ \overline{\mathrm{HOMO-5}}}^{\mathrm{LUMO+4}\ \overline{\mathrm{LUMO+4}}}$	0.25
		$\Phi_{\mathrm{HOMO-5}\ \overline{\mathrm{HOMO-5}}}^{\overline{\mathrm{LUMO+4}}\ \mathrm{LUMO+4}}$	0.25
		$\Phi_{\overline{\mathrm{HOMO-5}}\ \mathrm{HOMO-5}}^{\mathrm{LUMO+4}\ \overline{\mathrm{LUMO+4}}}$	0.25
		$\Phi_{\overline{\mathrm{HOMO-5}}\ \mathrm{HOMO-5}}^{\overline{\mathrm{LUMO+4}}\ \mathrm{LUMO+4}}$	0.25
		$\Phi_{\mathrm{HOMO-10}}^{\mathrm{LUMO}}$	0.11
		$\Phi_{\overline{\mathrm{HOMO-10}}}^{\overline{\mathrm{LUMO}}}$	0.11
T_1	3.1884	$\Phi_{\mathrm{HOMO}}^{\mathrm{LUMO}}$	48.9
		$\Phi_{\overline{\mathrm{HOMO}}}^{\overline{\mathrm{LUMO}}}$	48.9
		$\Phi_{\mathrm{HOMO}}^{\mathrm{LUMO+8}}$	1.0
		$\Phi_{\overline{\mathrm{HOMO}}}^{\overline{\mathrm{LUMO+8}}}$	1.0
S_1	3.4484	$\Phi_{\mathrm{HOMO}}^{\mathrm{LUMO}}$	48.8
		$\Phi_{\overline{\mathrm{HOMO}}}^{\overline{\mathrm{LUMO}}}$	48.8
		$\Phi_{\mathrm{HOMO-1}}^{\mathrm{LUMO+5}}$	1.1
		$\Phi_{\overline{\mathrm{HOMO-1}}}^{\overline{\mathrm{LUMO+5}}}$	1.1
T_2	3.5194	$\Phi_{\mathrm{HOMO}}^{\mathrm{LUMO+5}}$	17.3
		$\Phi_{\overline{\mathrm{HOMO}}}^{\overline{\mathrm{LUMO+5}}}$	17.3

$\Phi_{\mathrm{HOMO-1}}^{\mathrm{LUMO}}$	16.1
$\Phi_{\overline{\mathrm{HOMO-1}}}^{\overline{\mathrm{LUMO}}}$	16.1
$\Phi_{\mathrm{HOMO-2}}^{\mathrm{LUMO}}$	3.8
$\Phi_{\overline{\mathrm{HOMO-2}}}^{\overline{\mathrm{LUMO}}}$	3.8
$\Phi_{\mathrm{HOMO}}^{\mathrm{LUMO+7}}$	3.3
$\Phi_{\overline{\mathrm{HOMO}}}^{\overline{\mathrm{LUMO+7}}}$	3.3
$\Phi_{\mathrm{HOMO-3}}^{\mathrm{LUMO+7}}$	2.5
$\Phi_{\overline{\mathrm{HOMO-3}}}^{\overline{\mathrm{LUMO+7}}}$	2.5
$\Phi_{\mathrm{HOMO-1}}^{\mathrm{LUMO+6}}$	2.4
$\Phi_{\overline{\mathrm{HOMO-1}}}^{\overline{\mathrm{LUMO+6}}}$	2.4
$\Phi_{\mathrm{HOMO-9}}^{\mathrm{LUMO+8}}$	1.5
$\Phi_{\overline{\mathrm{HOMO-9}}}^{\overline{\mathrm{LUMO+8}}}$	1.5
$\Phi_{\mathrm{HOMO-4}}^{\mathrm{LUMO+9}}$	1.3
$\Phi_{\overline{\mathrm{HOMO-4}}}^{\overline{\mathrm{LUMO+9}}}$	1.3
$\Phi_{\mathrm{HOMO}}^{\mathrm{LUMO+9}}$	1.2
$\Phi_{\overline{\mathrm{HOMO}}}^{\overline{\mathrm{LUMO+9}}}$	1.2

Supplementary Table 7. S_0, S_1, T_1, and T_2 of DABNA-1 calculated at the EOM-CCSD/6-31G level of theory. The optimized S_1 geometry in Supplementary Table 2 was used. Φ_0 denotes the Hartree-Fock determinant. Φ_a^r and Φ_{ab}^{rs} denote single and double excitations from Φ_0, respectively.

State	Excitation energy (eV)	Slater determinant	Contribution (%)
S_0		Φ_0	98.04
		$\Phi_{\mathrm{HOMO-1}}^{\mathrm{LUMO}}$	0.32
		$\Phi_{\overline{\mathrm{HOMO-1}}}^{\overline{\mathrm{LUMO}}}$	0.32
		$\Phi_{\mathrm{HOMO-5}\ \overline{\mathrm{HOMO-5}}}^{\mathrm{LUMO+4}\ \overline{\mathrm{LUMO+4}}}$	0.26
		$\Phi_{\mathrm{HOMO-5}\ \overline{\mathrm{HOMO-5}}}^{\overline{\mathrm{LUMO+4}}\ \mathrm{LUMO+4}}$	0.26
		$\Phi_{\overline{\mathrm{HOMO-5}}\ \mathrm{HOMO-5}}^{\mathrm{LUMO+4}\ \overline{\mathrm{LUMO+4}}}$	0.26
		$\Phi_{\overline{\mathrm{HOMO-5}}\ \mathrm{HOMO-5}}^{\overline{\mathrm{LUMO+4}}\ \mathrm{LUMO+4}}$	0.26
		$\Phi_{\mathrm{HOMO-10}}^{\mathrm{LUMO}}$	0.11
		$\Phi_{\overline{\mathrm{HOMO-10}}}^{\overline{\mathrm{LUMO}}}$	0.11
T_1	3.0509	$\Phi_{\mathrm{HOMO}}^{\mathrm{LUMO}}$	48.7
		$\Phi_{\overline{\mathrm{HOMO}}}^{\overline{\mathrm{LUMO}}}$	48.7
		$\Phi_{\mathrm{HOMO}}^{\mathrm{LUMO+8}}$	1.2
		$\Phi_{\overline{\mathrm{HOMO}}}^{\overline{\mathrm{LUMO+8}}}$	1.2
S_1	3.2983	$\Phi_{\mathrm{HOMO}}^{\mathrm{LUMO}}$	48.8
		$\Phi_{\overline{\mathrm{HOMO}}}^{\overline{\mathrm{LUMO}}}$	48.8
		$\Phi_{\mathrm{HOMO-1}}^{\mathrm{LUMO+5}}$	1.1
		$\Phi_{\overline{\mathrm{HOMO-1}}}^{\overline{\mathrm{LUMO+5}}}$	1.1
T_2	3.4324	$\Phi_{\mathrm{HOMO-1}}^{\mathrm{LUMO}}$	18.2
		$\Phi_{\overline{\mathrm{HOMO-1}}}^{\overline{\mathrm{LUMO}}}$	18.2

$\Phi_{\mathrm{HOMO}}^{\mathrm{LUMO+5}}$	15.9
$\Phi_{\overline{\mathrm{HOMO}}}^{\overline{\mathrm{LUMO+5}}}$	15.9
$\Phi_{\mathrm{HOMO}}^{\mathrm{LUMO+7}}$	5.0
$\Phi_{\overline{\mathrm{HOMO}}}^{\overline{\mathrm{LUMO+7}}}$	5.0
$\Phi_{\mathrm{HOMO-2}}^{\mathrm{LUMO}}$	2.6
$\Phi_{\overline{\mathrm{HOMO-2}}}^{\overline{\mathrm{LUMO}}}$	2.6
$\Phi_{\mathrm{HOMO}}^{\mathrm{LUMO+9}}$	2.3
$\Phi_{\overline{\mathrm{HOMO}}}^{\overline{\mathrm{LUMO+9}}}$	2.3
$\Phi_{\mathrm{HOMO-3}}^{\mathrm{LUMO+7}}$	2.1
$\Phi_{\overline{\mathrm{HOMO-3}}}^{\overline{\mathrm{LUMO+7}}}$	2.1
$\Phi_{\mathrm{HOMO-1}}^{\mathrm{LUMO+6}}$	2.1
$\Phi_{\overline{\mathrm{HOMO-1}}}^{\overline{\mathrm{LUMO+6}}}$	2.1
$\Phi_{\mathrm{HOMO-9}}^{\mathrm{LUMO+8}}$	1.4
$\Phi_{\overline{\mathrm{HOMO-9}}}^{\overline{\mathrm{LUMO+8}}}$	1.4

Supplementary Table 8. S_0, S_1, T_1, and T_2 of DABNA-1 calculated at the EOM-CCSD/6-31G level of theory. The optimized T_1 geometry in Supplementary Table 3 was used. Φ_0 denotes the Hartree-Fock determinant. Φ_a^r and Φ_{ab}^{rs} denote single and double excitations from Φ_0, respectively.

State	Excitation energy (eV)	Slater determinant	Contribution (%)
S_0		Φ_0	98.04
		$\Phi_{\mathrm{HOMO-1}}^{\mathrm{LUMO}}$	0.32
		$\Phi_{\overline{\mathrm{HOMO-1}}}^{\overline{\mathrm{LUMO}}}$	0.32
		$\Phi_{\mathrm{HOMO-5}\ \overline{\mathrm{HOMO-5}}}^{\mathrm{LUMO+4}\ \overline{\mathrm{LUMO+4}}}$	0.26
		$\Phi_{\mathrm{HOMO-5}\ \overline{\mathrm{HOMO-5}}}^{\overline{\mathrm{LUMO+4}}\ \mathrm{LUMO+4}}$	0.26
		$\Phi_{\overline{\mathrm{HOMO-5}}\ \mathrm{HOMO-5}}^{\mathrm{LUMO+4}\ \overline{\mathrm{LUMO+4}}}$	0.26
		$\Phi_{\overline{\mathrm{HOMO-5}}\ \mathrm{HOMO-5}}^{\overline{\mathrm{LUMO+4}}\ \mathrm{LUMO+4}}$	0.26
		$\Phi_{\mathrm{HOMO-10}}^{\mathrm{LUMO}}$	0.12
		$\Phi_{\overline{\mathrm{HOMO-10}}}^{\overline{\mathrm{LUMO}}}$	0.12
T_1	3.0582	$\Phi_{\mathrm{HOMO}}^{\mathrm{LUMO}}$	48.8
		$\Phi_{\overline{\mathrm{HOMO}}}^{\overline{\mathrm{LUMO}}}$	48.8
		$\Phi_{\mathrm{HOMO}}^{\mathrm{LUMO+8}}$	1.1
		$\Phi_{\overline{\mathrm{HOMO}}}^{\overline{\mathrm{LUMO+8}}}$	1.1
S_1	3.3177	$\Phi_{\mathrm{HOMO}}^{\mathrm{LUMO}}$	48.8
		$\Phi_{\overline{\mathrm{HOMO}}}^{\overline{\mathrm{LUMO}}}$	48.8
		$\Phi_{\mathrm{HOMO-1}}^{\mathrm{LUMO+5}}$	1.1
		$\Phi_{\overline{\mathrm{HOMO-1}}}^{\overline{\mathrm{LUMO+5}}}$	1.1
T_2	3.4531	$\Phi_{\mathrm{HOMO-1}}^{\mathrm{LUMO}}$	19.5
		$\Phi_{\overline{\mathrm{HOMO-1}}}^{\overline{\mathrm{LUMO}}}$	19.5

	$\Phi_{\mathrm{HOMO}}^{\mathrm{LUMO+5}}$	14.4
	$\Phi_{\overline{\mathrm{HOMO}}}^{\overline{\mathrm{LUMO+5}}}$	14.4
	$\Phi_{\mathrm{HOMO}}^{\mathrm{LUMO+7}}$	4.8
	$\Phi_{\overline{\mathrm{HOMO}}}^{\overline{\mathrm{LUMO+7}}}$	4.8
	$\Phi_{\mathrm{HOMO-2}}^{\mathrm{LUMO}}$	3.4
	$\Phi_{\overline{\mathrm{HOMO-2}}}^{\overline{\mathrm{LUMO}}}$	3.4
	$\Phi_{\mathrm{HOMO-3}}^{\mathrm{LUMO+7}}$	2.4
	$\Phi_{\overline{\mathrm{HOMO-3}}}^{\overline{\mathrm{LUMO+7}}}$	2.4
	$\Phi_{\mathrm{HOMO-1}}^{\mathrm{LUMO+6}}$	2.1
	$\Phi_{\overline{\mathrm{HOMO-1}}}^{\overline{\mathrm{LUMO+6}}}$	2.1
	$\Phi_{\mathrm{HOMO}}^{\mathrm{LUMO+9}}$	1.6
	$\Phi_{\overline{\mathrm{HOMO}}}^{\overline{\mathrm{LUMO+9}}}$	1.6
	$\Phi_{\mathrm{HOMO-9}}^{\mathrm{LUMO+8}}$	1.5
	$\Phi_{\overline{\mathrm{HOMO-9}}}^{\overline{\mathrm{LUMO+8}}}$	1.5

Supplementary Table 9. S_0, S_1, T_1, and T_2 of DABNA-1 calculated at the EOM-CCSD/6-31G level of theory. The optimized T_2 geometry in Supplementary Table 4 was used. Φ_0 denotes the Hartree-Fock determinant. Φ_a^r and Φ_{ab}^{rs} denote single and double excitations from Φ_0, respectively.

State	Excitation energy (eV)	Slater determinant	Contribution (%)
S_0		Φ_0	98.06
		$\Phi_{\mathrm{HOMO-1}}^{\mathrm{LUMO}}$	0.32
		$\Phi_{\overline{\mathrm{HOMO-1}}}^{\overline{\mathrm{LUMO}}}$	0.32
		$\Phi_{\mathrm{HOMO-5}\ \overline{\mathrm{HOMO-5}}}^{\mathrm{LUMO+4}\ \overline{\mathrm{LUMO+4}}}$	0.26
		$\Phi_{\mathrm{HOMO-5}\ \overline{\mathrm{HOMO-5}}}^{\overline{\mathrm{LUMO+4}}\ \mathrm{LUMO+4}}$	0.26
		$\Phi_{\overline{\mathrm{HOMO-5}}\ \mathrm{HOMO-5}}^{\mathrm{LUMO+4}\ \overline{\mathrm{LUMO+4}}}$	0.26
		$\Phi_{\overline{\mathrm{HOMO-5}}\ \mathrm{HOMO-5}}^{\overline{\mathrm{LUMO+4}}\ \mathrm{LUMO+4}}$	0.26
		$\Phi_{\mathrm{HOMO-10}}^{\mathrm{LUMO}}$	0.11
		$\Phi_{\overline{\mathrm{HOMO-10}}}^{\overline{\mathrm{LUMO}}}$	0.11
T_1	3.1108	$\Phi_{\mathrm{HOMO}}^{\mathrm{LUMO}}$	49.2
		$\Phi_{\overline{\mathrm{HOMO}}}^{\overline{\mathrm{LUMO}}}$	49.2
		$\Phi_{\mathrm{HOMO}}^{\mathrm{LUMO+8}}$	0.7
		$\Phi_{\overline{\mathrm{HOMO}}}^{\overline{\mathrm{LUMO+8}}}$	0.7
S_1	3.3509	$\Phi_{\mathrm{HOMO}}^{\mathrm{LUMO}}$	48.7
		$\Phi_{\overline{\mathrm{HOMO}}}^{\overline{\mathrm{LUMO}}}$	48.7
		$\Phi_{\mathrm{HOMO-1}}^{\mathrm{LUMO+5}}$	1.2
		$\Phi_{\overline{\mathrm{HOMO-1}}}^{\overline{\mathrm{LUMO+5}}}$	1.2
T_2	3.3632	$\Phi_{\mathrm{HOMO-1}}^{\mathrm{LUMO}}$	28.4
		$\Phi_{\overline{\mathrm{HOMO-1}}}^{\overline{\mathrm{LUMO}}}$	28.4

$\Phi_{\mathrm{HOMO}}^{\mathrm{LUMO+5}}$	6.4
$\Phi_{\overline{\mathrm{HOMO}}}^{\overline{\mathrm{LUMO+5}}}$	6.4
$\Phi_{\mathrm{HOMO-3}}^{\mathrm{LUMO+7}}$	4.1
$\Phi_{\overline{\mathrm{HOMO-3}}}^{\overline{\mathrm{LUMO+7}}}$	4.1
$\Phi_{\mathrm{HOMO}}^{\mathrm{LUMO+7}}$	4.1
$\Phi_{\overline{\mathrm{HOMO}}}^{\overline{\mathrm{LUMO+7}}}$	4.1
$\Phi_{\mathrm{HOMO-3}}^{\mathrm{LUMO+2}}$	1.8
$\Phi_{\overline{\mathrm{HOMO-3}}}^{\overline{\mathrm{LUMO+2}}}$	1.8
$\Phi_{\mathrm{HOMO-1}}^{\mathrm{LUMO+6}}$	1.7
$\Phi_{\overline{\mathrm{HOMO-1}}}^{\overline{\mathrm{LUMO+6}}}$	1.7
$\Phi_{\overline{\mathrm{HOMO-2}}}^{\overline{\mathrm{LUMO}}}$	1.7
$\Phi_{\overline{\mathrm{HOMO-2}}}^{\overline{\mathrm{LUMO}}}$	1.7
$\Phi_{\mathrm{HOMO-9}}^{\mathrm{LUMO+8}}$	1.4
$\Phi_{\overline{\mathrm{HOMO-9}}}^{\overline{\mathrm{LUMO+8}}}$	1.4

Supplementary Table 10. CCSD total energies of S_0, and S_0-S_n, S_0-T_n excitation energies (n = 1, 2, 3, 4) of DABNA-1 calculated at the EOM-CCSD/6-31G level of theory. The S_0 energies are in hartree. The excitation energies are in eV.

	S_0 geometry	S_1 geometry	T_1 geometry	T_2 geometry
S_0	-1284.49691555	-1284.49381062	-1284.49482879	-1284.49472712
S_0-S_1	3.4484	3.2983	3.3177	3.3509
S_0-S_2	4.4293	4.3175	4.3319	4.2405
S_0-S_3	4.5524	4.4801	4.4713	4.5218
S_0-S_4	4.8762	4.8201	4.8260	4.8154
S_0-T_1	3.1884	3.0509	3.0582	3.1108
S_0-T_2	3.5194	3.4324	3.4531	3.3632
S_0-T_3	3.7094	3.6351	3.6463	3.5616
S_0-T_4	3.9125	3.7817	3.8156	3.8358

Supplementary Table 11. S_0, S_n, and T_n electronic energies (n = 1, 2, 3, 4) of DABNA-1 calculated from Supplementary Table 10. The zero point of the energies is set to be the S_0 energy at the S_0 geometry. The smallest S_0, S_1, T_1, and T_2 energies are underlined. The smallest S_0 energy is found at the S_0 geometry. The smallest S_1 and T_1 energies are found at the T_1 geometry. The smallest T_2 energy is found at the T_2 geometry. Thus, the local minima of the S_1 and T_1 potentials are located at the T_1 geometry. Values are in eV.

	S_0 geometry	S_1 geometry	T_1 geometry	T_2 geometry
S_0	**<u>0.0000</u>**	0.0845	0.0568	0.0596
S_1	3.4484	3.3828	**<u>3.3745</u>**	3.4105
S_2	4.4293	4.4020	4.3887	4.3001
S_3	4.5524	4.5646	4.5281	4.5814
S_4	4.8762	4.9046	4.8828	4.8750
T_1	3.1884	3.1354	**<u>3.1150</u>**	3.1704
T_2	3.5194	3.5169	3.5099	**<u>3.4228</u>**
T_3	3.7094	3.7196	3.7031	3.6212
T_4	3.9125	3.8662	3.8724	3.8954

Supplementary Table 12. S_0, S_n, and T_n electronic energies (n = 1, 2, 3, 4) of DABNA-1 after T_1 energy correction. The zero point of the energies is set to be the S_0 energy at the S_0 geometry. The smallest S_0, S_1, T_1, and T_2 energies are underlined. The smallest S_0 energy is found at the S_0 geometry. The smallest S_1 and T_1 energies are found at the T_1 geometry. The smallest T_2 energy is found at the T_2 geometry. Thus, the local minima of the S_1 and T_1 potentials are located at the T_1 geometry. Values are in eV.

	S_0 geometry	S_1 geometry	T_1 geometry	T_2 geometry
S_0	**<u>0.00</u>**	0.08	0.06	0.06
S_1	3.45	3.38	**<u>3.37</u>**	3.41
S_2	4.43	4.40	4.39	4.30
S_3	4.55	4.56	4.53	4.58
S_4	4.88	4.90	4.88	4.88
T_1	3.25	3.20	**<u>3.18</u>**	3.23
T_2	3.52	3.52	3.51	**<u>3.42</u>**
T_3	3.71	3.72	3.70	3.62
T_4	3.91	3.87	3.87	3.90

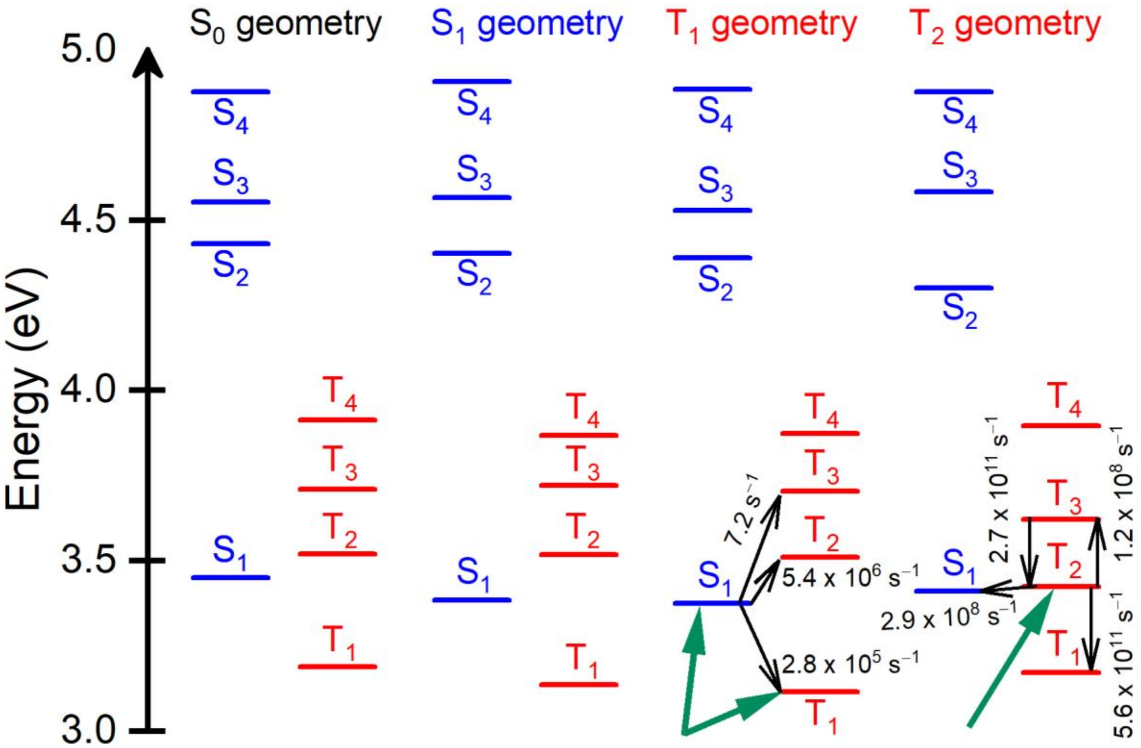

Supplementary Figure 3. Energy level diagrams calculated at the EOM-CCSD/6-31G level of theory. The minimum S_1, T_1, and T_2 energies are designated by green arrows. Electronic transitions from/to T_3 are designated by black arrows. The numerical values at the black arrows are the corresponding rate constants.

Supplementary Table 13. Energy difference between electronic states (meV).

	S_0 geom	S_1T_1	T_2 geom
Raw EOM-CCSD (Supplementary Table 11)			
$\Delta E(S_1\text{-}T_2)$	71	135	12
$\Delta E(T_1\text{-}T_2)$	331	395	242
$\Delta E(S_1\text{-}T_1)$	260	259	240
Corrected (Supplementary Table 12)			
$\Delta E(S_1\text{-}T_2)$	71	135	12
$\Delta E(T_1\text{-}T_2)$	271	335	182
$\Delta E(S_1\text{-}T_1)$	200	199	180

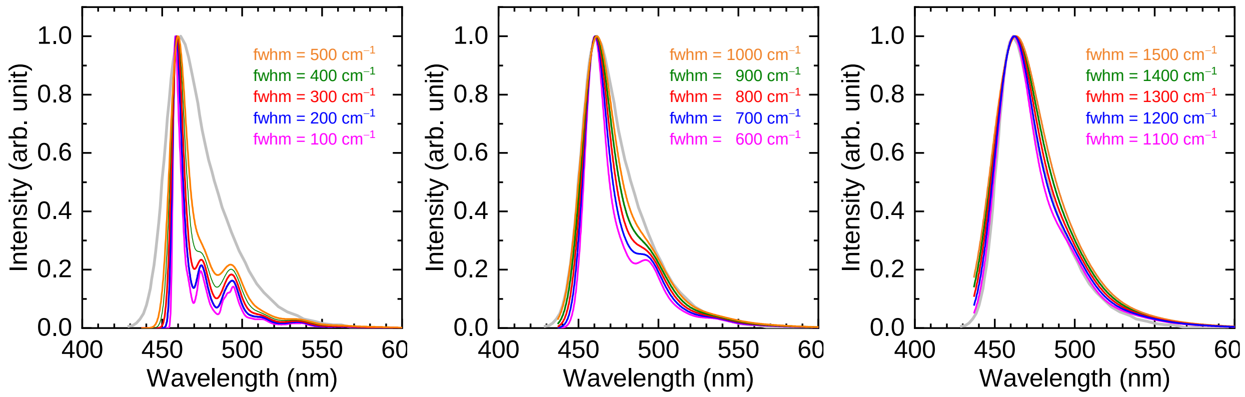

Supplementary Figure 4. Calculated (the orange, green, red, blue, and purple lines) and experimental (the grey lines) emission spectra in CH_2Cl_2 solution. The emission spectra were calculated at the TD-TPSSh/6-31+G(d) level of theory with fwhm value of 100–1500 cm^{-1}. The best fit between the calculated and experimental spectra was obtained when fwhm = 1200 cm^{-1}, where fwhm = 2γ (see Supplementary Equation S4).

Supplementary Table 14. Interstate vibronic-coupling ($V_\alpha(T_m\text{-}T_n)@T_2$) and the resulting $R_\alpha/\hbar$ of the αth vibrational mode between S_0 and S_1 for DABNA-1 calculated at the T_2 geometry. R_α are calculated using the corrected $\Delta E(T_1\text{-}T_2)$. The line shape function (LSF) for the αth vibrational mode. Contribution from the αth mode to k_{IC} ($k_{IC,\,\alpha}$). $k_{IC} = \Sigma_\alpha\, k_{IC,\,\alpha} = 5.63\times10^{11}$ s^{-1}. $R_\alpha/\hbar$, $LSF_{IC,\,\alpha}$, and $r_{IC,\,\alpha}$ versus wavenumber plots are shown in Supplementary Figure 5.

α	Wavenumber (cm^{-1})	V_α (10^{-4} a.u.)	$R_\alpha/\hbar$ (10^{14} s^{-1})	$LSF_{IC,\,\alpha}$ (10^{-3})	$k_{IC,\,\alpha}$ (10^{11} s^{-1})
1	21.81	0.023066	0.000044	2.640700	0.000116
2	22.23	0.085906	0.000610	2.591100	0.001581
3	41.64	0.153510	0.001949	1.391300	0.002711
4	47.64	0.003319	0.000001	1.219000	0.000001
5	53.07	0.005090	0.000002	1.097100	0.000002
6	73.84	0.016978	0.000024	0.798310	0.000019
7	74.31	0.010583	0.000009	0.793530	0.000007
8	79.34	0.129710	0.001391	0.745800	0.001038
9	90.75	0.227960	0.004297	0.657860	0.002827
10	119.56	0.000734	0.000000	0.513240	0.000000
11	169.91	0.015420	0.000020	0.384780	0.000008
12	184.93	3.649100	1.101200	0.361520	0.398090
13	202.62	0.020537	0.000035	0.339380	0.000012
14	204.47	0.408020	0.013767	0.337330	0.004644
15	205.88	0.145530	0.001751	0.335810	0.000588
16	208.95	1.281200	0.135740	0.332570	0.045144
17	234.38	0.008272	0.000006	0.309980	0.000002
18	265.37	0.005966	0.000003	0.290280	0.000001
19	276.46	1.218600	0.122800	0.284820	0.034976
20	295.33	2.786700	0.642180	0.277080	0.177940
21	337.81	1.385800	0.158810	0.265310	0.042134
22	359.49	0.697130	0.040189	0.261660	0.010516
23	380.56	0.037872	0.000119	0.259330	0.000031
24	406.27	0.068563	0.000389	0.257870	0.000100
25	409.25	0.952810	0.075074	0.257790	0.019353
26	414.00	0.001314	0.000000	0.257690	0.000000
27	417.00	0.613720	0.031147	0.257660	0.008025
28	420.25	0.027014	0.000060	0.257630	0.000016
29	449.41	0.121110	0.001213	0.258250	0.000313
30	449.60	0.707990	0.041451	0.258250	0.010705
31	461.01	1.144000	0.108230	0.258870	0.028016
32	501.18	0.664110	0.036472	0.262490	0.009573
33	508.28	0.010085	0.000008	0.263350	0.000002
34	521.59	0.022842	0.000043	0.265120	0.000011
35	525.06	0.814420	0.054850	0.265620	0.014569
36	534.17	0.431900	0.015426	0.266990	0.004118
37	575.17	0.330580	0.009037	0.274270	0.002479

38	592.32	0.000612	0.000000	0.277830	0.000000
39	610.31	0.000649	0.000000	0.281880	0.000000
40	616.74	0.447380	0.016551	0.283400	0.004691
41	621.92	0.037955	0.000119	0.284660	0.000034
42	621.96	0.004676	0.000002	0.284670	0.000001
43	625.60	0.000675	0.000000	0.285570	0.000000
44	640.30	0.000042	0.000000	0.289320	0.000000
45	645.67	0.603850	0.030153	0.290740	0.008767
46	676.23	0.284750	0.006705	0.299330	0.002007
47	684.11	0.011770	0.000011	0.301680	0.000003
48	688.19	0.147470	0.001798	0.302920	0.000545
49	705.58	0.007767	0.000005	0.308390	0.000002
50	706.15	1.103700	0.100730	0.308570	0.031084
51	721.26	0.004273	0.000002	0.313550	0.000000
52	725.56	0.009917	0.000008	0.315000	0.000003
53	725.80	0.016782	0.000023	0.315080	0.000007
54	740.33	0.130140	0.001401	0.320130	0.000448
55	743.22	6.743300	3.760300	0.321150	1.207600
56	761.57	0.802630	0.053273	0.327850	0.017466
57	768.09	0.007892	0.000005	0.330310	0.000002
58	789.37	2.597800	0.558070	0.338600	0.188960
59	800.22	0.004179	0.000001	0.343000	0.000000
60	816.02	0.840370	0.058401	0.349610	0.020417
61	839.66	0.001180	0.000000	0.359960	0.000000
62	856.82	0.000452	0.000000	0.367830	0.000000
63	856.90	0.064657	0.000346	0.367860	0.000127
64	857.99	0.001483	0.000000	0.368370	0.000000
65	864.36	0.722410	0.043156	0.371380	0.016027
66	872.14	0.004129	0.000001	0.375110	0.000001
67	894.96	0.003572	0.000001	0.386420	0.000000
68	899.45	1.833300	0.277930	0.388720	0.108040
69	919.45	0.000071	0.000000	0.399210	0.000000
70	929.19	0.503410	0.020957	0.404480	0.008477
71	942.25	0.070902	0.000416	0.411730	0.000171
72	942.52	0.000297	0.000000	0.411880	0.000000
73	946.16	0.392340	0.012729	0.413940	0.005269
74	947.05	0.003193	0.000001	0.414440	0.000000
75	956.34	1.149000	0.109170	0.419770	0.045828
76	987.56	0.000453	0.000000	0.438480	0.000000
77	987.57	0.008801	0.000006	0.438480	0.000003
78	1008.10	0.043816	0.000159	0.451470	0.000072
79	1008.10	0.000011	0.000000	0.451470	0.000000
80	1012.30	0.002236	0.000000	0.454190	0.000000
81	1012.60	0.822840	0.055990	0.454390	0.025441
82	1041.90	0.214300	0.003798	0.474080	0.001800

83	1047.20	0.000272	0.000000	0.477780	0.000000
84	1050.60	0.870800	0.062707	0.480170	0.030110
85	1052.80	0.000567	0.000000	0.481720	0.000000
86	1079.60	0.001308	0.000000	0.501250	0.000000
87	1100.00	0.030361	0.000076	0.516850	0.000039
88	1100.10	0.122360	0.001238	0.516920	0.000640
89	1102.40	1.660700	0.228060	0.518720	0.118300
90	1111.20	2.408900	0.479860	0.525690	0.252260
91	1112.30	0.019520	0.000032	0.526570	0.000017
92	1137.90	0.000614	0.000000	0.547610	0.000000
93	1171.90	0.002058	0.000000	0.577290	0.000000
94	1187.60	0.000044	0.000000	0.591690	0.000000
95	1187.60	0.016257	0.000022	0.591690	0.000013
96	1189.70	0.002834	0.000001	0.593660	0.000000
97	1191.80	2.404000	0.477910	0.595620	0.284650
98	1198.50	1.454900	0.175040	0.601960	0.105370
99	1199.00	0.010811	0.000010	0.602440	0.000006
100	1204.80	0.476710	0.018793	0.608000	0.011426
101	1223.30	3.298600	0.899780	0.626180	0.563420
102	1233.20	0.002138	0.000000	0.636180	0.000000
103	1247.90	1.943300	0.312290	0.651400	0.203420
104	1248.70	0.002774	0.000001	0.652240	0.000000
105	1272.80	0.000373	0.000000	0.678190	0.000000
106	1292.30	0.650660	0.035009	0.700090	0.024510
107	1313.50	0.000534	0.000000	0.724830	0.000000
108	1324.60	0.412490	0.014070	0.738180	0.010386
109	1340.30	0.000228	0.000000	0.757530	0.000000
110	1340.60	0.033456	0.000093	0.757910	0.000070
111	1362.10	0.118540	0.001162	0.785340	0.000913
112	1362.20	0.000008	0.000000	0.785470	0.000000
113	1363.70	0.485260	0.019473	0.787430	0.015333
114	1367.30	0.001437	0.000000	0.792140	0.000000
115	1392.20	0.189290	0.002963	0.825540	0.002446
116	1416.40	0.098988	0.000810	0.859380	0.000696
117	1427.90	0.000471	0.000000	0.875940	0.000000
118	1443.60	0.000614	0.000000	0.899040	0.000000
119	1456.60	0.391900	0.012701	0.918590	0.011667
120	1477.50	0.311670	0.008033	0.950820	0.007638
121	1483.10	0.000772	0.000000	0.959620	0.000000
122	1488.10	0.012783	0.000014	0.967530	0.000013
123	1488.30	0.036785	0.000112	0.967850	0.000108
124	1495.30	2.348000	0.455900	0.979020	0.446340
125	1506.40	0.000940	0.000000	0.996940	0.000000
126	1519.50	0.981420	0.079650	1.018400	0.081116
127	1531.90	0.005986	0.000003	1.039000	0.000003

128	1532.30	0.736420	0.044846	1.039700	0.046627
129	1548.80	0.000799	0.000000	1.067600	0.000000
130	1565.30	0.003067	0.000001	1.095900	0.000001
131	1577.10	2.874200	0.683140	1.116500	0.762710
132	1632.30	0.003947	0.000001	1.214600	0.000002
133	1632.40	0.005083	0.000002	1.214700	0.000003
134	1639.60	0.000550	0.000000	1.227700	0.000000
135	1640.70	0.499410	0.020625	1.229700	0.025362
136	3185.60	0.937490	0.072679	0.339200	0.024653
137	3186.40	0.000101	0.000000	0.338840	0.000000
138	3191.40	0.005969	0.000003	0.336570	0.000001
139	3191.40	0.155610	0.002002	0.336570	0.000674
140	3194.20	0.000012	0.000000	0.335320	0.000000
141	3198.40	0.000357	0.000000	0.333440	0.000000
142	3198.40	0.009796	0.000008	0.333440	0.000003
143	3203.40	0.458480	0.017383	0.331230	0.005758
144	3205.50	0.000014	0.000000	0.330300	0.000000
145	3206.50	0.428540	0.015187	0.329870	0.005010
146	3206.50	0.005673	0.000003	0.329870	0.000001
147	3212.30	0.378540	0.011849	0.327340	0.003879
148	3212.80	0.001981	0.000000	0.327120	0.000000
149	3212.80	0.015074	0.000019	0.327120	0.000006
150	3219.50	0.001228	0.000000	0.324240	0.000000
151	3219.60	0.393650	0.012814	0.324190	0.004154
152	3224.90	0.000047	0.000000	0.321940	0.000000
153	3230.30	0.855770	0.060561	0.319660	0.019359
154	3231.90	1.351800	0.151110	0.318990	0.048204
155	3232.40	0.001352	0.000000	0.318780	0.000000
156	3234.10	0.000056	0.000000	0.318070	0.000000

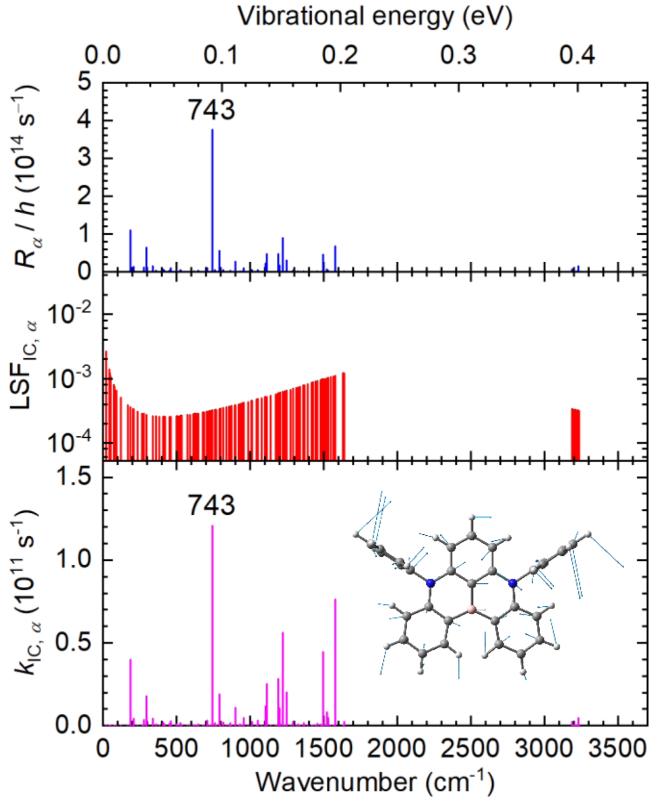

Supplementary Figure 5. Calculated $R_\alpha/\hbar$, $LSF_{IC,\,\alpha}$, and $k_{IC,\,\alpha}$ for the $T_1@T_2 \rightarrow T_2@T_2$ internal conversion of DABNA-1. The vibrational mode with the wavenumber of 743 cm^{-1} has the largest contribution to the $T_1@T_2 \rightarrow T_2@T_2$ internal conversion. The inset figure shows the vibrational mode with the wavenumber of 743 cm^{-1}.

The kinetic equations for calculating S_0, S_1, T_1, and T_2 populations

$$\frac{d}{dt}[\mathrm{S_1@S_1T_1}](t) = -\left(k_{\mathrm{S_1\to T_1}}^{\mathrm{S_1T_1}} + k_{\mathrm{S_1\to T_2}}^{\mathrm{S_1T_1}} + k_{\mathrm{S_1\to S_0}}^{\mathrm{S_1T_1}} + k_{\mathrm{F}}^{\mathrm{S_1T_1}}\right)[\mathrm{S_1@S_1T_1}](t)$$
$$+k_{\mathrm{T_1\to S_1}}^{\mathrm{S_1T_1}}[\mathrm{T_1@S_1T_1}](t) + k_{\mathrm{T_2\to S_1}}^{\mathrm{S_1T_1}}[\mathrm{T_2@S_1T_1}](t) + k_{\mathrm{S_1,GR}}[\mathrm{S_1@T_2}](t) \quad \text{(S10)}$$

$$\frac{d}{dt}[\mathrm{T_1@S_1T_1}](t) = -\left(k_{\mathrm{T_1\to S_1}}^{\mathrm{S_1T_1}} + k_{\mathrm{T_1\to T_2}}^{\mathrm{S_1T_1}} + k_{\mathrm{P,T_1}}^{\mathrm{S_1T_1}}\right)[\mathrm{T_1@S_1T_1}](t)$$
$$+k_{\mathrm{S_1\to T_1}}^{\mathrm{S_1T_1}}[\mathrm{S_1@S_1T_1}](t) + k_{\mathrm{T_2\to T_1}}^{\mathrm{S_1T_1}}[\mathrm{T_2@S_1T_1}](t) + k_{\mathrm{T_1,GR}}[\mathrm{T_1@T_2}](t) \quad \text{(S11)}$$

$$\frac{d}{dt}[\mathrm{T_2@S_1T_1}](t) = -\left(k_{\mathrm{T_2\to S_1}}^{\mathrm{S_1T_1}} + k_{\mathrm{T_2\to T_1}}^{\mathrm{S_1T_1}} + k_{\mathrm{T_2\to S_0}}^{\mathrm{S_1T_1}} + k_{\mathrm{P,T_2}}^{\mathrm{S_1T_1}} + k_{\mathrm{T_2,GR}}\right)[\mathrm{T_2@S_1T_1}](t)$$
$$+k_{\mathrm{S_1\to T_2}}^{\mathrm{S_1T_1}}[\mathrm{S_1@S_1T_1}](t) + k_{\mathrm{T_1\to T_2}}^{\mathrm{S_1T_1}}[\mathrm{T_1@S_1T_1}](t) \quad \text{(S12)}$$

$$\frac{d}{dt}[\mathrm{S_0@S_1T_1}](t) = \left(k_{\mathrm{S_1\to S_0}}^{\mathrm{S_1T_1}} + k_{\mathrm{F}}^{\mathrm{S_1T_1}}\right)[\mathrm{S_1@S_1T_1}](t)$$
$$+\left(k_{\mathrm{T_1\to S_0}}^{\mathrm{S_1T_1}} + k_{\mathrm{P,T_1}}^{\mathrm{S_1T_1}}\right)[\mathrm{T_1@S_1T_1}](t) + \left(k_{\mathrm{T_2\to S_0}}^{\mathrm{S_1T_1}} + k_{\mathrm{P,T_2}}^{\mathrm{S_1T_1}}\right)[\mathrm{T_2@S_1T_1}](t) \quad \text{(S13)}$$

$$\frac{d}{dt}[\mathrm{S_1@T_2}](t) = -\left(k_{\mathrm{S_1\to T_1}}^{\mathrm{T_2}} + k_{\mathrm{S_1\to T_2}}^{\mathrm{T_2}} + k_{\mathrm{S_1\to S_0}}^{\mathrm{T_2}} + k_{\mathrm{F}}^{\mathrm{T_2}} + k_{\mathrm{S_1,GR}}\right)[\mathrm{S_1@T_2}](t)$$

$$+k_{\mathrm{T_1\to S_1}}^{\mathrm{T_2}}[\mathrm{T_1@T_2}](t) + k_{\mathrm{T_2\to S_1}}^{\mathrm{T_2}}[\mathrm{T_2@T_2}](t) \quad \text{(S14)}$$

$$\frac{d}{dt}[\mathrm{T_1@T_2}](t) = -\left(k_{\mathrm{T_1\to S_1}}^{\mathrm{T_2}} + k_{\mathrm{T_1\to T_2}}^{\mathrm{T_2}} + k_{\mathrm{P,T_1}}^{\mathrm{T_2}} + k_{\mathrm{T_1,GR}}\right)[\mathrm{T_1@T_2}](t)$$

$$+k_{\mathrm{S_1\to T_1}}^{\mathrm{T_2}}[\mathrm{S_1@T_2}](t) + k_{\mathrm{T_2\to T_1}}^{\mathrm{T_2}}[\mathrm{T_2@T_2}](t) + k_{\mathrm{T_1,GD}}[\mathrm{T_1@S_1T_1}](t) \quad \text{(S15)}$$

$$\frac{d}{dt}[\mathrm{T_2@T_2}](t) = -\left(k_{\mathrm{T_2\to S_1}}^{\mathrm{T_2}} + k_{\mathrm{T_2\to T_1}}^{\mathrm{T_2}} + k_{\mathrm{T_2\to S_0}}^{\mathrm{T_2}} + k_{\mathrm{P,T_2}}^{\mathrm{T_2}}\right)[\mathrm{T_2@T_2}](t)$$

$$+k_{\mathrm{S_1\to T_2}}^{\mathrm{T_2}}[\mathrm{S_1@T_2}](t) + k_{\mathrm{T_1\to T_2}}^{\mathrm{T_2}}[\mathrm{T_1@T_2}](t) + k_{\mathrm{T_2,GR}}[\mathrm{T_2@S_1T_1}](t) \quad \text{(S16)}$$

$$\frac{d}{dt}[\mathrm{S_0@T_2}](t) = \left(k_{\mathrm{S_1\to S_0}}^{\mathrm{T_2}} + k_{\mathrm{F}}^{\mathrm{T_2}}\right)[\mathrm{S_1@T_2}](t) + \left(k_{\mathrm{T_1\to S_0}}^{\mathrm{T_1}} + k_{\mathrm{P,T_1}}^{\mathrm{T_1}}\right)[\mathrm{T_1@T_2}](t)$$

$$+\left(k_{\mathrm{T_2\to S_0}}^{\mathrm{T_2}} + k_{\mathrm{P,T_2}}^{\mathrm{T_2}}\right)[\mathrm{T_2@T_2}](t) \quad \text{(S17)}$$

Supplementary Table 15. Definitions of the variables and constants in Eqs S10–17.

$[S_0@S_1T_1]$	the population of S_0 at the S_1T_1 geometry
$[S_1@S_1T_1]$	the population of S_1 at the S_1T_1 geometry
$[T_1@S_1T_1]$	the population of T_1 at the S_1T_1 geometry
$[T_2@S_1T_1]$	the population of T_2 at the S_1T_1 geometry
$[S_0@T_2]$	the population of S_0 at the T_2 geometry
$[S_1@T_2]$	the population of S_1 at the T_2 geometry
$[T_1@T_2]$	the population of T_1 at the T_2 geometry
$[T_2@T_2]$	the population of T_2 at the T_2 geometry
$k_{S_1 \to T_1}^{S_1T_1}$	the rate constant for the $S_1 \to T_1$ nonradiative transition at the S_1T_1 geometry
$k_{S_1 \to T_2}^{S_1T_1}$	the rate constant for the $S_1 \to T_2$ nonradiative transition at the S_1T_1 geometry
$k_{S_1 \to S_0}^{S_1T_1}$	the rate constant for the $S_1 \to S_0$ nonradiative decay at the S_1T_1 geometry
$k_{F}^{S_1T_1}$	the rate constant for the $S_1 \to S_0$ radiative decay at the S_1T_1 geometry
$k_{T_1 \to S_1}^{S_1T_1}$	the rate constant for the $T_1 \to S_1$ nonradiative transition at the S_1T_1 geometry
$k_{T_2 \to S_1}^{S_1T_1}$	the rate constant for the $T_2 \to S_1$ nonradiative transition at the S_1T_1 geometry
$k_{T_1 \to T_2}^{S_1T_1}$	the rate constant for the $T_1 \to T_2$ nonradiative transition at the S_1T_1 geometry
$k_{P,T_1}^{S_1T_1}$	the rate constant for the $T_1 \to S_0$ radiative decay at the S_1T_1 geometry
$k_{T_2 \to T_1}^{S_1T_1}$	the rate constant for the $T_2 \to T_1$ nonradiative transition at the S_1T_1 geometry
$k_{T_2 \to S_0}^{S_1T_1}$	the rate constant for the $T_2 \to S_0$ nonradiative decay at the S_1T_1 geometry
$k_{P,T_2}^{S_1T_1}$	the rate constant for the $T_2 \to S_0$ radiative decay at the S_1T_1 geometry
$k_{S_1 \to T_1}^{T_2}$	the rate constant for the $S_1 \to T_1$ nonradiative transition at the T_2 geometry
$k_{S_1 \to T_2}^{T_2}$	the rate constant for the $S_1 \to T_2$ nonradiative transition at the T_2 geometry
$k_{S_1 \to S_0}^{T_2}$	the rate constant for the $S_1 \to S_0$ nonradiative decay at the T_2 geometry

$k_{F}^{T_2}$	the rate constant for the $S_1 \rightarrow S_0$ radiative decay at the T_2 geometry
$k_{T_1 \rightarrow S_1}^{T_2}$	the rate constant for the $T_1 \rightarrow S_1$ nonradiative transition at the T_2 geometry
$k_{T_2 \rightarrow S_1}^{T_2}$	the rate constant for the $T_2 \rightarrow S_1$ nonradiative transition at the T_2 geometry
$k_{T_1 \rightarrow T_2}^{T_2}$	the rate constant for the $T_1 \rightarrow T_2$ nonradiative transition at the T_2 geometry
$k_{P,T_1}^{T_2}$	the rate constant for the $T_1 \rightarrow S_0$ radiative decay at the T_2 geometry
$k_{T_2 \rightarrow T_1}^{T_2}$	the rate constant for the $T_2 \rightarrow T_1$ nonradiative transition at the T_2 geometry
$k_{T_2 \rightarrow S_0}^{T_2}$	the rate constant for the $T_2 \rightarrow S_0$ nonradiative decay at the T_2 geometry
$k_{P,T_2}^{T_2}$	the rate constant for the $T_2 \rightarrow S_0$ radiative decay at the T_2 geometry
$k_{S_1,GR}$	the rate constant for the geometry relaxation in S_1
$k_{T_1,GR}$	the rate constant for the geometry relaxation in T_1
$k_{T_2,GR}$	the rate constant for the geometry relaxation in T_2
$k_{T_1,GD}$	the rate constant for the energy-uphill geometry deformation in T_1

Supplementary Note 1

Assumptions in the kinetic equations

First, $k_{ISC}(T_1 \rightarrow S_0)$ is set to be zero. Second, because the calculated energy difference between $T_1@S_1T_1$ and $T_1@T_2$ (55 meV) is sufficiently small, the $T_1@S_1T_1 \rightarrow T_1@T_2$ conversion occurs more frequently than the competitive $T_1 \rightarrow S_0$ decays. Hence, the energy-uphill $T_1@S_1T_1 \rightarrow T_1@T_2$ conversion owing to geometry deformation (GD) is assumed to be allowed. Here, we examine the validity of this assumption. GD in T_1 competes with phosphorescence. If the GD rate constant (k_{GD}) is much larger than k_p (~0.2 s^{-1}), GD occurs before T_1 decays to S_0. k_{GD} can be expressed as $k_{GD} = k_{GR} \times \exp(-\Delta E(T_1)/k_BT)$, where k_{GR} is the rate constant for geometry relaxation, $\Delta E(T_1)$ = 55 meV is the energy difference between $T_1@S_1T_1$ and $T_1@T_2$ (Figure 1b), k_B is the Boltzmann constant, and T is the temperature. Even if k_{GR} is as small as 1.00×10^3 s^{-1}, $k_{GD} = 1.17\times10^2$ s^{-1} and $k_{GD} >> k_P$. k_{GR} of organic molecules is typically 10^{12} s^{-1} and hence, it is reasonable to assume that $k_{GD} >> k_P$ holds for DABNA-1. Here, k_{GR} is set to be 1.00×10^{12} s^{-1} and $k_{GD} = 1.17\times10^{11}$ s^{-1}.

Supplementary References

1 Frisch, M. J. *et al.* Gaussian 16 Rev. C.01. (2016)

2 Shao, Y. *et al.* Advances in molecular quantum chemistry contained in the Q-Chem 4 program package. *Mol. Phys.* **113**, 184-215 (2015).
